# A radically simple, ingestible colorimetric biosensor pill for cost-effective, non-invasive monitoring of intestinal inflammation

**DOI:** 10.1016/j.device.2025.100865

**Published:** 2025-11-21

**Authors:** Zile Zhuang, Lucia L. Huang, Bo Chan Seo, Subhashini Pandey, Jeffrey M. Karp, Yuhan Lee, Caitlin L. Maikawa

**Affiliations:** 1 Department of Anesthesiology, Perioperative and Pain Medicine, Center for Accelerated Medical Innovation & Center for Nanomedicine, Brigham and Women’s Hospital, Boston, MA 02115, USA; 2 Harvard Medical School, Boston, MA 02115, USA; 3 Harvard–MIT Division of Health Sciences and Technology, Cambridge, MA 02139, USA; 4 Proteomics Platform, Broad Institute, Cambridge, MA 02142, USA; 5 Harvard Stem Cell Institute, Cambridge, MA 02138, USA; 6 Institute of Biomedical Engineering, University of Toronto, Toronto, ON M5S3E3, Canada; 7 Department of Chemistry, University of Toronto, Toronto, ON M5S3H6, Canada; 8 These authors contributed equally; 9 Lead contact

## Abstract

Inflammatory bowel diseases (IBDs) affect millions worldwide, necessitating frequent monitoring of intestinal inflammation to optimize treatment strategies. However, current fecal calprotectin tests have low patient adherence, limiting their utility for inflammation monitoring. Here, we developed an ingestible biosensor for simplified at-home detection of a key inflammation biomarker—reactive oxygen species (ROS). Our pill for ROS-responsive inflammation monitoring (PRIM) employs an ROS-responsive polymer that selectively degrades in the presence of ROS. Degradation triggers the release of blue dye into feces for a visually detectable readout without fecal sampling or laboratory analysis. *In vitro*, PRIM remained stable under healthy conditions and activated only at elevated ROS levels (10–50 mM H_2_O_2_). In rats with colitis, the miniaturized PRIM demonstrated a sensitivity of 78% and a specificity of 72% in detecting intestinal inflammation. With further optimization, PRIM has the potential to improve accessibility and patient adherence to inflammation monitoring and enhance personalized disease management for IBD.

## In brief

This study describes the development of an ingestible pill-based biosensor device, the pill for reactive oxygen species (ROS)-responsive inflammation monitoring (PRIM), that uses ROS-responsive polymers to trigger colored dye release in the presence of intestinal inflammation. Proof of concept is supported by consistent observation of blue feces in rats with colitis. The PRIM device’s simple dye release mechanism would eliminate fecal sample handling and analysis and is a promising candidate for a patient-friendly, cost-effective solution for intestinal inflammation monitoring.

## Graphical Abstract

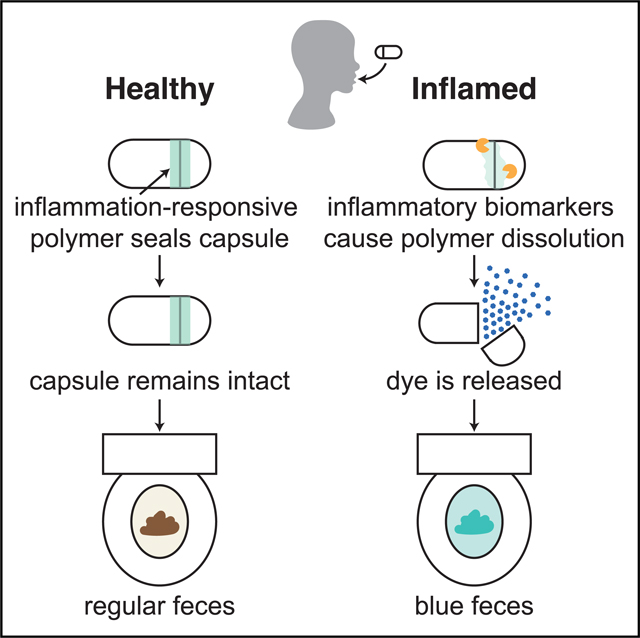

## INTRODUCTION

Inflammatory bowel diseases (IBDs), such as Crohn’s disease and ulcerative colitis, affect over 7 million patients worldwide. IBD has the highest incidence rate in the United States, Western Europe, and Canada, but there is rising prevalence in newly industrialized countries in Asia, the Middle East, and South America.^1,2^ IBD is episodic and is characterized by uncontrolled inflammation primarily in the small intestine and colon.^3–5^ There is no cure for IBD, but there are effective treatments available that can be used to help patients reach and maintain remission.^6,7^ During periods of active inflammation, patients experience symptoms such as abdominal pain, diarrhea, bloody stool, weight loss, and fatigue that lower their quality of life.^3,4^ Control and prevention of inflammatory flare-ups are important, because poorly managed IBD leads to symptom flares and incidents requiring hospitalization and surgery. Current clinical practice guidelines advise an approach called treat-to-target, which recommends that the goal of medical therapies is to attain complete healing of the intestinal mucosa.^8,9^ One study observed a 50% decrease in hospitalizations with this tighter control of inflammation.^10^ However, symptoms cannot reliably predict mucosal healing in IBD, and many IBD patients exhibit no clinical symptoms despite endoscopic findings of inflammation and tissue damage.^11,12^ Thus, objective monitoring strategies are required to inform treatment regimens and enable patients to achieve mucosal healing.^10,13^

Present options for inflammation monitoring remain suboptimal. The gold standard for assessing mucosal healing in IBD is to perform a colonoscopy and collect intestinal tissue samples for histological analysis. While colonoscopy will remain a critical part of IBD diagnosis and disease monitoring, the invasiveness, requirement for specialized medical personnel and facilities, patient discomfort, and high cost make colonoscopy unviable for the frequent disease monitoring required for short-term and intermediate-term targets in the treat-to-target approach.^14^ Other, less invasive diagnostic methods exist but have limited use in monitoring. For example, computed tomographic enterography (CTE), magnetic resonance imaging (MRI), and ultrasonography are non-invasive but require a visit to a healthcare provider and are relatively insensitive to mild-to-moderate inflammation levels. Studies show that indirect measures of mucosal healing, like intestinal inflammation as measured by fecal biomarkers (e.g., fecal calprotectin), correlate well to colonoscopy findings, and a decrease in fecal calprotectin levels is considered an intermediate target under the Selecting Therapeutic Targets in Inflammatory Bowel Disease (STRIDE)-II guidelines for IBD treatment.^14^ Fecal testing is more accessible for patients, enabling patients to collect samples at home and mail them in for laboratory analysis. However, these tests have limited real-world usefulness for regular monitoring because only 35%–54% of patients return prescribed tests, in part because of unwillingness to collect stool samples.^3,15–17^ Recent studies have developed pH-sensitive, photosensitive, electrode-based, and bacterial-sensor-based pill devices to monitor changes in the gastrointestinal (GI) environment associated with inflammation.^18–21^ However, these devices remain complex, requiring electrochemical or genetic circuits for sensing, which reduces the likelihood of clinical adoption and increases costs for patients. A simple, accessible, patient-administered monitoring device that eliminates the need for fecal sampling or handling could facilitate widespread adoption for frequent inflammation monitoring in IBD patients, enabling objective and personalized disease management strategies.

**Figure 1.**
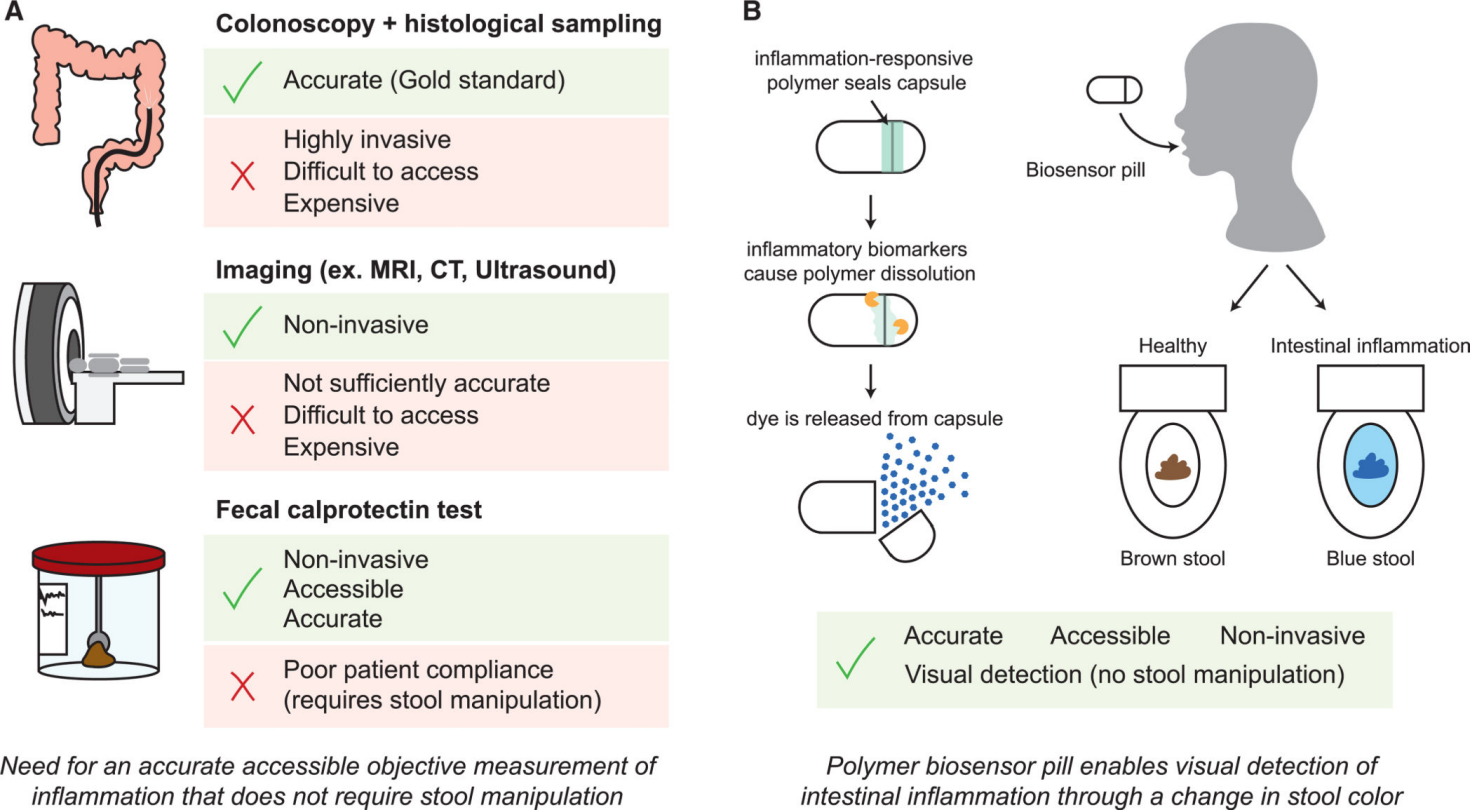
Schematic of modalities for inflammation monitoring in IBD (A) Current inflammation-monitoring techniques include (1) colonoscopy with histological sampling, (2) imaging modalities (e.g., magnetic resonance imaging [MRI], computed tomography [CT], ultrasound), and (3) fecal calprotectin testing. However, these techniques are all suboptimal for regular monitoring. (B) Pill-based inflammation monitoring that provides a visually detectable readout without fecal sample manipulation would be accessible and easy to use for patients. Our approach uses an inflammation-responsive polymer that acts to seal a pill capsule loaded with colored dye. Upon exposure to inflammatory biomarkers, polymer dissolution occurs, and dye is released from the pill capsule. The dye colors feces blue and is also released from fecal matter into the toilet bowl for easy detection.

To address this gap, we developed a Pill for Reactive oxygen species (ROS)-responsive Inflammation Monitoring (PRIM)—a radically simple, ingestible biosensor designed to provide a non-invasive, at-home method for detecting intestinal inflammation in a patient-friendly and cost-effective manner (Figure 1). Regular monitoring using the PRIM device would provide valuable data to clinicians for treatment decisions. The PRIM device leverages ROS, such as hydrogen peroxide (H_2_O_2_), as an indicator of intestinal inflammation. ROS are emerging as a biomarker for inflammation detection.^22^ ROS play critical roles in repair and immunity, and it is well established that ROS are an integral factor in chronic GI inflammation. Both fecal calprotectin and H_2_O_2_ production have been associated with neutrophil activation and infiltration into the gut mucosa, an early and persistent sign of IBD.^23^ Under normal conditions, ROS levels in the intestinal lumen are tightly regulated by anti-oxidant pathways.^22^ However, during chronic inflammation, excess ROS are produced from multiple sources, including increased immune cell activity, mitochondrial dysfunction in intestinal epithelial cells, and an imbalance of the gut microbiome, which contributes to tissue damage.^22,24–26^ Accordingly, increases in ROS levels are found to correlate with active IBD inflammation and can increase by 10–100 times.^22,24–26^ Notably, past studies have shown increased ROS production in both inflamed and healthy tissues, suggesting that their levels may be elevated throughout the GI tract during inflammatory flares, even in endoscopically normal tissues.^25^ Despite this, the transient nature of H_2_O_2_ and other ROS have made it difficult for existing technologies to detect and quantify them. The biological half-life of H_2_O_2_ is estimated to be around 0.31 min,^27^ making measurement in clinical samples (i.e., fecal samples) ineffective. Thus, to use H_2_O_2_ as a biomarker for intestinal inflammation, it must typically be measured locally in the intestines using an ingested chemical probe or device that produces a more stable readout.^28^

The advantage of ROS over fecal calprotectin for IBD monitoring is that there are a number of established ROS-responsive polymer chemistries that trigger physical changes in polymer materials including degradation, changes in solubility, and changes in fluorescence that can be used to develop simple biosensor devices.^29–31^ These polymer materials exploit ROS-responsive functional groups such as arylboronic esters that are rapidly oxidized and are preferentially cleaved in the presence of certain ROS, like H_2_O_2_.^32–34^ Thus, using ROS as a biomarker has potential to enable a materials-based inflammation sensing platform that will measure local levels of ROS in the intestines.

Using this approach, PRIM integrates an ROS-responsive dextran polymer as a sealing mechanism in a capsule-based biosensor. The polymer degrades selectively under elevated H_2_O_2_ levels, triggering the release of a blue dye as a visual indicator of inflammation. Here, we demonstrate that the PRIM device remains stable under healthy conditions but activates in response to elevated H_2_O_2_ levels ranging from 10 to 50 mM. In a rat model of acute colitis, we demonstrate that a miniaturized PRIM device detects intestinal inflammation by dyeing fecal samples blue. By eliminating the need for direct fecal handling and laboratory analysis, PRIM offers a cost-effective, patient-friendly alternative for routine monitoring with the potential to improve compliance and facilitate personalized disease management.

## RESULTS

**Figure 2.**
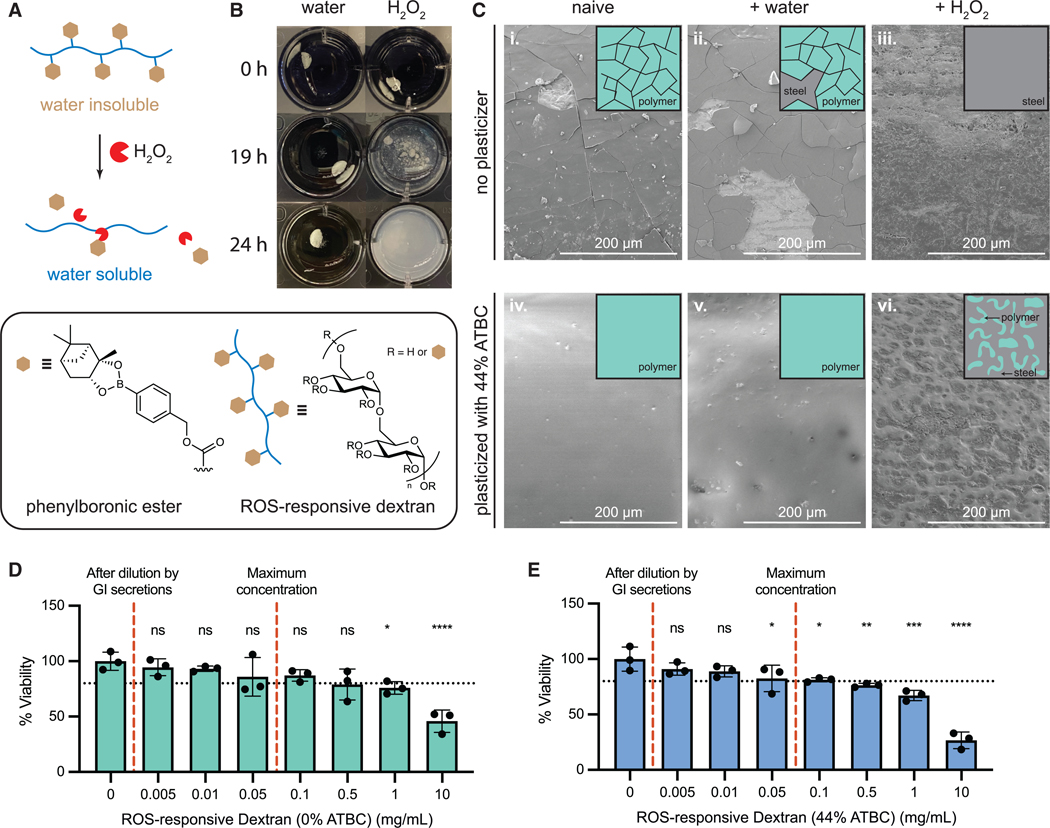
Solubility switch of ROS-responsive dextran polymer (A) Schematic of degradation of ROS-responsive dextran. Conjugation of hydrophobic phenylboronic esters to dextran results in a water-insoluble polymer. Exposure to H_2_O_2_ cleaves the phenylboronic esters to restore dextran’s water solubility. (B) ROS-responsive dextran degrades and dissolves following exposure to <100 mM H_2_O_2_ (right) but remains an insoluble solid in water (left). (C) Scanning electron microscopy (SEM) images of (i–iii) unplasticized ROS-responsive dextran and (iv–vi) ROS-responsive dextran plasticized with 44% w/w ATBC. Devices were (i and iv) freshly prepared, (ii and v) exposed to water for 4 h (ii) and 24 h (v), (iii and vi) exposed to 50 mM H_2_O_2_ for 4 h prior to imaging. (D and E) MTT assay to test cytotoxicity of ROS-responsive dextran (D), and ROS-responsive dextran with ATBC (E). Black dotted line shows the 80% threshold considered to be a healthy culture. Statistical significance was determined by one-way ANOVA. Dunnett *post hoc* tests were applied for multiple comparisons to the 0 mg/mL control (see Tables 1 and S2for numerical *p* values). Legend for (D) and (E): ns = not significant, *p < 0.05, **p < 0.01, ***p < 0.001, ****p < 0.0001. Data are represented as mean ± standard deviation.

Our PRIM device uses a materials approach to sense ROS that are upregulated during inflammation in the intestinal tract. To detect elevated ROS, we synthesized an ROS-responsive polymer by modifying dextran, a biocompatible polysaccharide, with phenylboronic ester functional groups (Figures 2A and S1–S5).^31^ While unmodified dextran is hydrophilic and water soluble, conjugation of hydrophobic phenylboronic ester moieties renders the polymer water insoluble. Phenylboronic esters are selectively degraded in the presence of H_2_O_2_^35^–a type of ROS upregulated in IBD patients^25,36^–and cleavage of these hydrophobic groups from the polymer restores the dextran’s water-soluble form. We demonstrate this ROS-responsive solubility switch in Figure 2B—when incubated in water, the ROS-responsive dextran is insoluble and remains as a solid piece. Exposure of the polymer to H_2_O_2_ (100 mM) resulted in disintegration of the polymer within 24 h, demonstrating the ROS responsiveness of the material.

Polymer coatings made from ROS-responsive dextran were visualized using scanning electron microscopy (SEM) to examine morphological differences between naive samples and samples exposed to water or H_2_O_2_ (Figure 2C). We note the presence of cracks in coatings made from purified ROS-responsive dextran (containing no plasticizer). Partial flaking of the brittle polymer layer was observed after exposure to water, while no polymer layer was observed after exposure to H_2_O_2_. We also observed that coatings made from ROS-responsive dextran containing unreacted phenylboronic ester (prior to polymer purification; Figures S4 and S5) were less brittle and exhibited fewer cracks compared to coatings made with purified polymer (Figure S6); thus, we hypothesized that residual phenylboronic ester can act as a plasticizer for the ROS-responsive dextran. Accordingly, to improve the mechanical properties of the purified ROS-responsive dextran, we incorporated 44% w/w acetyl tributyl citrate (ATBC) as an additive to the purified polymer—ATBC is a common biodegradable plasticizer that is used to shift the glass transition temperature of polymer materials.^37,38^ The addition of the ATBC plasticizer resulted in smooth coatings that remained intact even after exposure to water for 24 h (Figure 2C). After exposure to H_2_O_2_, coatings displayed a visibly eroded topography that confirmed retention of the polymer’s ROS-responsive properties after plasticizer addition—the textured regions represent sites where the polymer has been degraded by H_2_O_2_ and subsequently dissolved away. We also observed that the hydrophobic properties of ATBC were important for plasticizing functions, as more hydrophilic plasticizers, such as triethyl citrate, rapidly leached out, returning the polymer to a brittle state. Both the ROS-responsive dextran and ROS-responsive dextran with 44% w/w ATBC were also tested for potential cytotoxicity. Cell viability between 80% and 95% of the control cells is accepted to represent a healthy culture, and the mean cell viability of all polymer concentrations at or above the maximum concentration expected in the body (0.06 mg/mL) were above 80% (Figures 2D and 2E). Statistical multiple-comparison tests show that only polymer with ATBC at 0.05 mg/mL is statistically different from the control (Tables S1 and S2), but this is not highly concerning because (1) the mean (82.5%) is greater than 80%, (2) the majority of the polymer will be insoluble and trapped on the device, and (3) the polymer will be rapidly diluted by other GI fluids as it transits through the GI tract.

**Figure 3.**
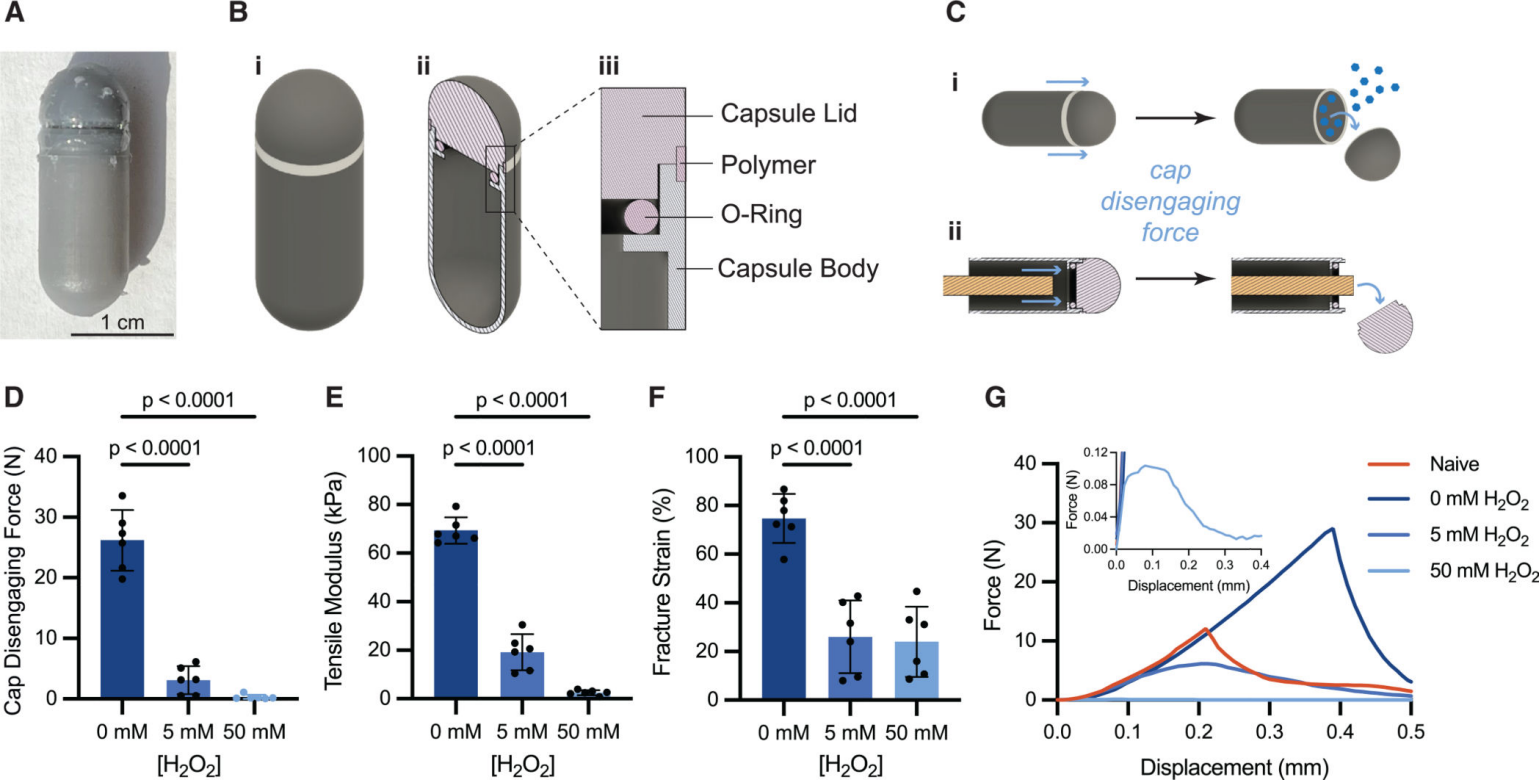
PRIM device schematics and mechanical testing (A) PRIM device sealed with ROS-responsive polymer adhesive coating. (B) Schematic of (i) the 00 capsule-sized PRIM device, (ii) cross-section of the PRIM device showing the capsule lid and the hollow chamber that is filled with dye, and (iii) inset of the cross-section showing how an O-ring is used to form a seal between the capsule body and lid and where ROS-responsive dextran is filled into a notch to adhere the lid and body together. (C) Schematic of (i) PRIM device mechanism design for cap detachment and dye release and (ii) experimental measurement of cap disengaging force using a rod to push off the cap. (D–F) (D) Cap disengaging force, (E) tensile modulus of polymer cap adhesive, and (F) tensile fracture strain after 24 h exposure to 0, 5, or 50 mM H_2_O_2_ (*n* = 6 devices per group, one experiment). Statistical significance was determined by one-way ANOVA. Dunnett *post hoc* tests were applied for multiple comparisons to the 0 mM control. Data are represented as mean ± standard deviation. (G) Example force-displacement curves from cap-detachment tests after device manufacture or after 24 h exposure to 0, 5, or 50 mM H_2_O_2_.

The PRIM device is designed to be similar in size to a 00 capsule (Figure 3A), a common size used for over-the-counter medication, and is composed of a 3D-printed pill cap and body sealed by an O-ring (Figures 3B and S7). The device body is filled with brilliant blue FCF, a commonly used food coloring that is not absorbed in the digestive tract and will cause changes in feces color when consumed in sufficient quantities.^39,40^ The junction between the device cap and body of the pill is designed with a notch for the addition of ROS-responsive dextran to adhere the cap and body together. Detachment of the cap upon exposure to ROS enables a simple, binary release of brilliant blue dye that indicates the presence of increased ROS levels. ROS-responsive biomaterials report performing *in vitro* testing in H_2_O_2_ concentrations ranging from 0.5 to 50 mM.^32,41–45^ This range scales with the 10- to 100-fold elevation in ROS levels expected with inflammation, and the physiologically relevant levels of H_2_O_2_ that have been reported in healthy individuals are 35–100 μM (plasma and urine, respectively).^22,46–50^ For our *in vitro* experiments we have selected 0, 1, 10, and 50 mM concentrations to be consistent with the range observed in the literature.

The ROS-responsive dextran, plasticized with 44% w/w ATBC, was used as an adhesive to seal the cap and body of our PRIM device. As the polymer degrades and dissolves in the presence of H_2_O_2_, the adhesive force weakens, significantly decreasing the force necessary to detach the cap from the device body (Figure 3C). The adhesive strength was measured as the force required to push the cap off the PRIM device (Figure 3D) using the experimental setup illustrated in Figures S8 and S9. As expected, we found that cap-disengagement force differed between devices exposed to H_2_O_2_ (F_2,15_ = 119.3, *p* < 0.0001). Cap-disengagement force after exposure to H_2_O_2_ for 24 h (5 mM H_2_O_2_: 3 ± 2 N, *p* < 0.0001. 50 mM H_2_O_2_: 0.3 ± 0.4 N, *p* < 0.0001) was lower compared to devices exposed to phosphate buffer without H_2_O_2_ (0 mM H_2_O_2_: 26 ± 5 N) for the same amount of time (Figure 3D). Similarly, the tensile modulus of the polymer differed between samples exposed to H_2_O_2_ (F_2,15_ = 253.2, *p* < 0.0001). Tensile modulus decreased after exposure to H_2_O_2_ for 24 h (5 mM H_2_O_2_: 19 ± 7 kPa, *p* < 0.0001. 50 mM H_2_O_2_: 2.5 ± 1 kPa, *p* < 0.0001. 0 mM H_2_O_2_: 69 ± 5 kPa), indicating that the polymer became more deformable under lower amounts of applied stress (Figure 3E). The H_2_O_2_-exposed polymer also underwent notable plastic deformation at low levels of strain (Figure 3G), and fracture strain differed after exposure to H_2_O_2_ (F_7,38_ = 253.2, *p* < 0.0001). Polymer samples exposed to H_2_O_2_ fractured at lower strain than polymer exposed to buffer for 24 h (5 mM H_2_O_2_: 26 ± 15%, *p* < 0.0001. 50 mM H_2_O_2_: 24 ± 14%, *p* < 0.0001. 0 mM H_2_O_2_: 75 ± 10%) (Figure 3F). These mechanical insights support a mechanism where cap detachment is facilitated by adhesive weakening after exposure to H_2_O_2_. Without H_2_O_2_, the polymer retains its adhesive strength even after 48 h (22 ± 5 N, t = 1.616, df = 10, *p* = 0.1372; Figure S10B); however, the fracture strain decreased (48 h: 54% ± 9%. 24 h: 75 ± 10%, t = 3.802, df = 10, *p* = 0.0035) (Figure S10H), which may result from plasticizer leaching over time. Interestingly, the strength of the ROS-responsive dextran increased following incubation in buffer without H_2_O_2_ compared to freshly coated samples (Figures S10A and S10B). We hypothesize that the intermolecular interactions in the hydrophobic polymer-plasticizer mixture increase upon exposure to aqueous environments, resulting in stronger adhesive forces—this is reflected in the increased work of adhesion, represented by area under the curve of the force-displacement plot (Figure 3G).

**Figure 4.**
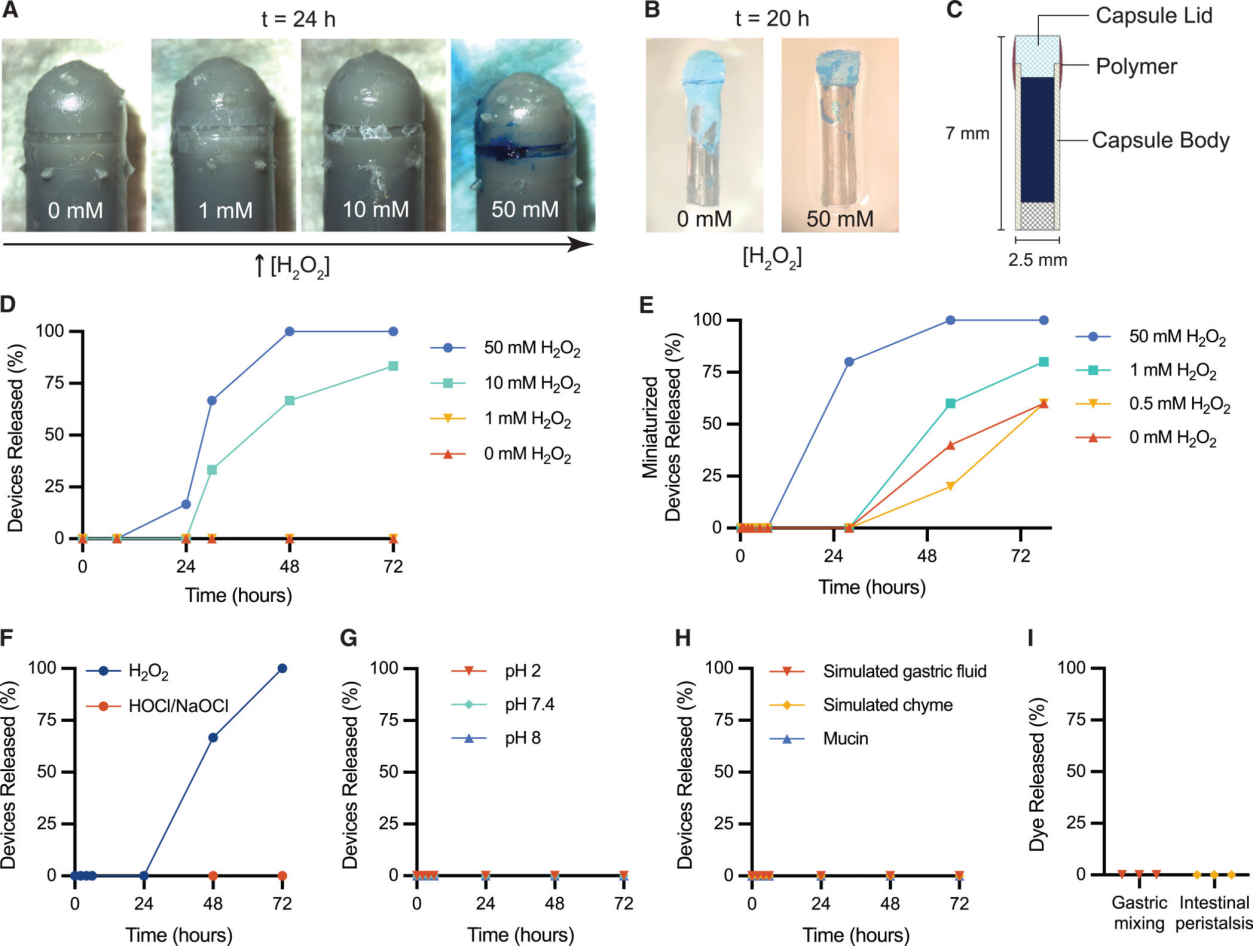
PRIM release and stability assays (A) PRIM after 24 h exposure in 0, 1, 10, or 50 mM H_2_O_2_. (B) Miniaturized PRIM devices after 20 h exposure to 0 or 50 mM H_2_O_2_. (C) Schematic of miniaturized PRIM device for size-9 rat capsules. (D and E) (D) *In vitro* release assay of the percentage of 00-sized device capsules that have released dye cargo over time in 0, 1, 10, or 50 mM H_2_O_2_ (*n* = 6 devices per group, one experiment) and (E) percentage of miniaturized devices that have released dye cargo over time in 0, 0.5, 1, or 50 mM H_2_O_2_ (*n* = 5 devices per group, one experiment). Device release is defined as >10% of loaded dye released. (F–H) *In vitro* stability tests showing the percentage of device capsules that have released dye cargo over time in (F) H_2_O_2_ (50 mM, pH 7.4) or HOCl/NaOCl (50 mM, pH 8), (G) phosphate buffer (10 mM; pH 2, 7.4, and 8), and (H) mucin (3% w/v), simulated gastric fluid (0.64% w/v pepsin, pH 1.2), and simulated chyme (applesauce) (*n* = 3 devices per group, one experiment). Device release is defined as >10% of loaded dye released. (I) Percentage of dye released from devices after simulated gastric mixing (2–3.5 h) in *ex vivo* porcine stomachs with simulated gastric fluid and simulated intestinal peristalsis (72 h) in sausage casings with simulated chyme.

The cap detachment creates a threshold mechanism for ROS-responsive release of dye cargo (brilliant blue FCF) from the PRIM device. In this design, elevated ROS levels during inflammation are sufficient to degrade the polymer adhesive and detach the cap, while healthy baseline ROS levels fall below the threshold, resulting in no release. At low concentrations of H_2_O_2_ (0–1 mM) *in vitro*, no devices released their cargo, even after 72 h (Figure 4D). In elevated H_2_O_2_ (50 mM) conditions, all devices experienced complete degradation of the polymer adhesive within 48 h (Figure S11B) and released their dye cargo within 72 h (Figures 4D and 4F), which is within the GI transit time frame in humans with IBD (10–103 h, median 44.5 h).^51,52^ In 10 mM H_2_O_2_, 66% of devices released the dye within 48 h and 83% of devices released their cargo within 72 h (Figure 4D). These release timelines are similar to observed total GI transit times in severe ulcerative colitis patients, which have a median transit time of 44.5 h^52^ Due to the threshold release mechanism, dye release was observed to be a binary response, with devices displaying either complete release or minimal release (<10% of loaded dye; Figures S11–S15). While it was expected that devices would have similar degradation rates and time to release, some differences were noted, likely resulting from minor variances in device manufacturing and polymer-coating processes, and these effects were exacerbated if H_2_O_2_ levels were near the threshold necessary for release. The response time could be adjusted by varying the polymer-coating method and the device design (Figures S16 and S17). A preliminary meshed-lid design demonstrated faster dye release, with 66% of devices releasing dye in under 8 h at 50 mM H_2_O_2_, compared to the original PRIM design, which achieved 66% device released in less than 30 h under the same conditions (Figures 4D and S17C). Similar to PRIM, no dye release is observed from the meshed-lid device in the absence of H_2_O_2_.

While these release assays effectively compare PRIM’s performance under various H_2_O_2_ concentrations, we note these simulated conditions may not represent the complexities of the GI environment. *In vitro*, stoichiometric amounts of H_2_O_2_ are scavenged and consumed as our ROS-responsive polymer degrades, which decreases the H_2_O_2_ concentration until the release medium is replaced at the next time point. Under cellular inflammatory conditions, ROS like H_2_O_2_ are constantly secreted—for example, cultured inflamed tissue from ulcerative colitis patients produced H_2_O_2_ at a rate of 1.11 mmol/min/mg protein *ex vivo* but is also degraded through enzymatic anti-oxidant pathways.^25^ These dynamic changes in ROS levels have been poorly characterized *in vivo* and are difficult to recreate in a static-release assay. Moreover, the presence of other upregulated ROS or reactive nitrogen species during inflammation *in vivo*, such as hypochlorous acid (HOCl) or peroxynitrite, may work in tandem with H_2_O_2_ to increase the sensitivity of the PRIM device. While no PRIM devices released dye after exposure to HOCl alone *in vitro* (Figure 4F), it was observed that the ROS-responsive polymer layer underwent notable degradation in the presence of HOCl (Figure S12C).

To evaluate the PRIM device’s stability, we studied its performance in various *in vitro* tests that model healthy GI environments, including variable pH conditions, digestive enzymes, and digestive mechanical forces. All devices remained intact after incubation in acidic (pH 2), neutral (pH 7.4), and alkaline conditions (pH 8) for 72 h (Figure 4G). Similarly, all devices remained intact after exposure to simulated mucus (mucin), simulated gastric fluid (pepsin in hydrochloric acid, pH 1.2), and simulated chyme (food pureé) for 72 h (Figure 4H). Furthermore, all devices remained intact following continuous mechanical agitation of the device at physiologically relevant timescales (Figures 4I, S18, and S19). Gastric mixing was modeled by mechanically squeezing and prodding *ex vivo* porcine stomachs filled with simulated gastric fluid for 2–3.5 h (Video S1), which is the average gastric emptying time after a meal.^53^ Intestinal peristalsis was modeled by rolling motions over sausage casings filled with simulated chyme for 72 h (Video S2). Based on these results, we expect the PRIM device to remain stable in non-inflamed physiological conditions at relevant timescales.

**Figure 5.**
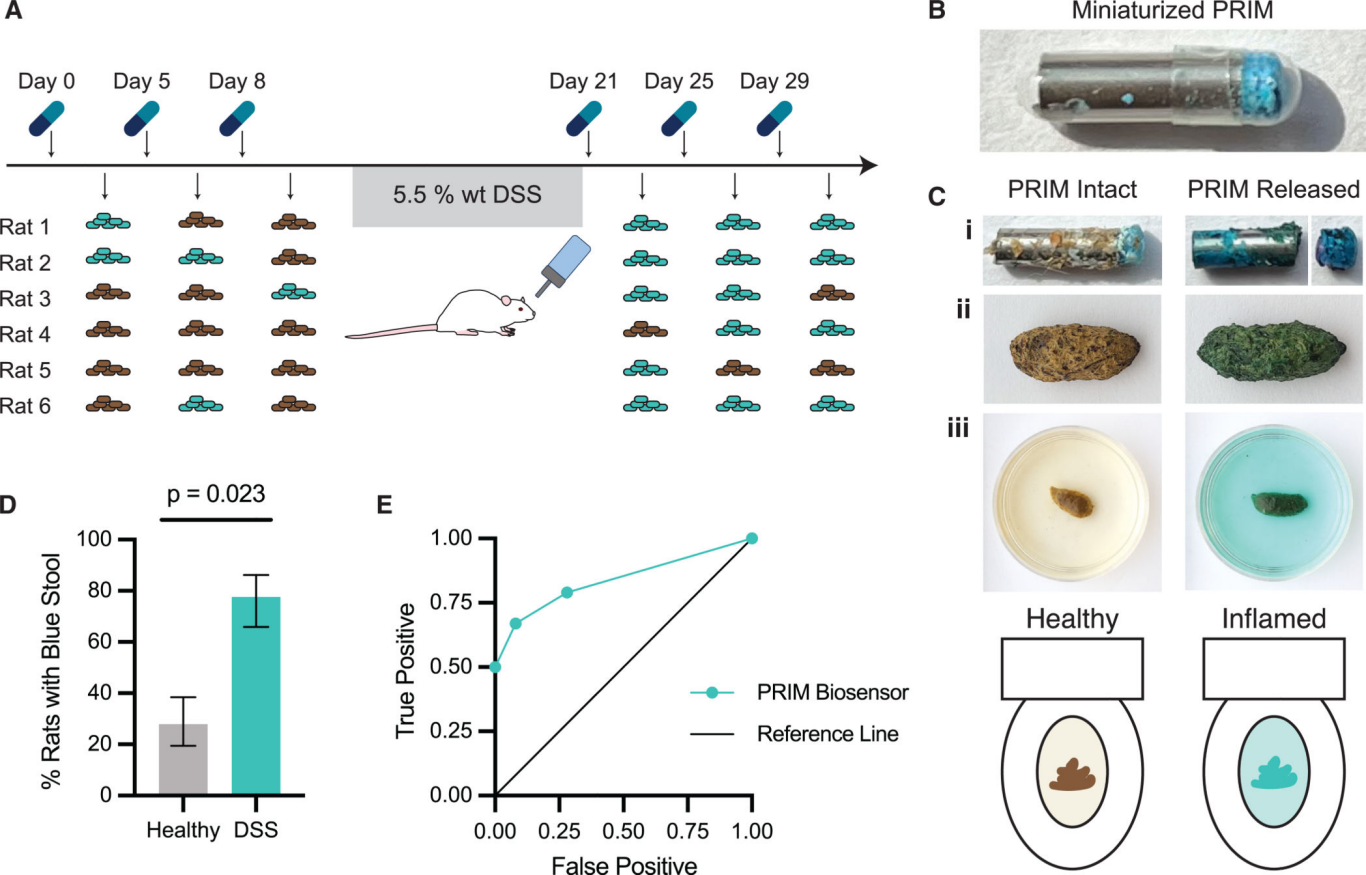
Detection of inflammation in rats following DSS administration using PRIM (A) Schematic of *in vivo* experimental setup and feces color. Studies were conducted using miniaturized PRIM devices (length = 7 mm) administered to a cohort of six Sprague-Dawley rats by oral gavage. Rat feces were collected up to 72 h after oral gavage and observed for color change. Colitis was induced using 5.5% w/w dextran sulfate sodium in drinking water. (B) Image of miniaturized PRIM device in capsule prior to administration. (C) Representative images of recovered devices and resulting feces color change. (i) Intact PRIM device (left) and activated device (right) recovered from rat stool samples. Device cap detaches upon administration. (ii) Activated devices cause feces color to shift from normal brown (left) to brilliant blue (right). (iii) Schematic and images of rat feces in water (after 15 min) simulating feces in a toilet bowl from healthy rat (left), and rat with DSS colitis (right). (D) Percentage of healthy or colitis rats with blue stool following device administration. Statistical significance was determined by conducting a generalized linear model with a logistic link function. Data are represented as mean ± standard error. (E) Receiver operating characteristic (ROC) curve of PRIM device for a single PRIM, two repeat administrations, and three repeat administrations.

To test the PRIM device *in vivo*, it needed to be miniaturized to enable oral administration in rats. PRIM was reduced to 7 mm in length and encapsulated in a standard size-9 capsule (Figures 4B, 4C, and 5B). The miniaturized device consisted of a steel-tube device body and punched-out plastic cap. ROS-responsive dextran was added to the device using dip coating. It should be noted that the miniaturized device was too small to include the notch for polymer application that was used on the full-sized PRIM device. Also, the ROS-responsive dextran used contained unreacted phenylboronic ester units rather than ATBC plasticizer. Similar to the full-sized PRIM, the devices demonstrate more rapid dye release when exposed to high levels of H_2_O_2_ (Figure 4E). However, the miniaturized device is less robust than the full-sized PRIM, and dye release is observed in low H_2_O_2_ concentrations after 48 h. Rats have a shorter GI transit time than humans, and it is expected that, even though there is a higher risk of false positives with the miniaturized devices after 36 h, typically devices will be cleared more quickly, and this will not significantly impact proof-of-concept testing.

To validate the PRIM platform *in vivo*, we miniaturized the device for oral gavage and tested its performance in a cohort of six rats using a dextran sulfate sodium (DSS) colitis rat model. Devices were first administered in healthy rats, where each rat received three devices. Colitis was then induced with DSS administered in drinking water for 7 days. Following the colitis induction period, each rat received another three devices. Stool consistency scoring was used to verify the onset of inflammation (0 = normal, 1 = soft, 2 = very soft, 3 = diarrhea; Figure S20). All rats reached stool scores of 2 during the DSS administration period, and the average stool score for the rat cohort during the device testing period (day 21–32) was 1.1 ± 0.5 (Figure S21).

When administered by oral gavage to healthy rats as a control, the majority of devices (13 devices, 72%) passed through the rats without activation, being eliminated in the feces and subsequently recovered, while five out of 18 (28%) devices activated releasing dye cargo (Figure 5A). No visual changes in feces color were detected when devices were passed intact (Figure 5C). In contrast, when PRIM devices were administered to rats with DSS-induced colitis, 14 out of 18 (78%) devices activated, with the lid separating from the capsule body (Figures 5A and 5C). Rats with activated devices produced feces of a bright blue color for 24–48 h (Figures 5C, S22, and S23). The change in feces color was transient, and feces color returned to normal, dark-brown colors after around 48 h. Our results demonstrate that PRIM devices selectively activate in response to established models of colitis (*p* = 0.023; Figure 5D) and cause an easily detectable visual change in feces color, with 78% specificity and 72% sensitivity (Figure 5E). There was no effect observed between PRIM dye release and device transit time (*p* = 0.81), which was approximated by the days until device recovery or the appearance of blue dye, whichever was observed first (Figure S24). The lack of a correlation between PRIM activation and time supports that the miniaturized PRIM is sufficiently mechanically stable for *in vivo* rat tests and that the observed dye release is a result of enhanced inflammation in colitis rats. Specificity of the PRIM device can be tuned through repeated device administrations—two sequential positive test results increased specificity to 92% (with 67% sensitivity), and three sequential positives provide 100% specificity (with 50% sensitivity) (Figure 5E). To test the impact of a variable diet that contains natural pigments, we co-administered brilliant blue FCF dye and betanin (beet dye) in a healthy rat. We observed no interference with the identification of the blue signal in the presence of betanin (Figure S25). Additionally, when blue feces are immersed in water, the dye diffuses out, causing the water to turn blue as well (Figure 5C). In clinical scenarios, this effect could aid the patient or provider in identifying device activation and increase sensitivity, for example, by observing water color changes in the toilet.

## DISCUSSION

In this study, we developed PRIM, an ingestible device for minimally invasive and accessible monitoring of elevated ROS biomarkers associated with inflammation in the GI tract. The PRIM device is sealed using an ROS-responsive dextran as a polymer adhesive, which selectively degrades upon exposure to H_2_O_2_ to release dye cargo loaded in the device. We demonstrate that the ROS-responsive adhesive is stable in the absence of H_2_O_2_ and that no dye is released when the device is exposed to low concentrations of H_2_O_2_. Furthermore, ROS-responsive dextran has been demonstrated to be a biocompatible polymer in cytotoxicity assays (Figure 2D). We also estimated the cost of production of the PRIM device at scale to be $0.38 per device (Table S3). When compared with estimates of other monitoring technologies (e.g., fecal calprotectin, wireless capsule endoscopy, experimental wireless capsule technologies), PRIM has the lowest cost and does not need specialized personnel or equipment to obtain a result (Table S4).

When administered to DSS-colitis rats, our miniaturized PRIM device successfully indicated the presence of intestinal inflammation by releasing brilliant blue FCF dye, resulting in visibly colored fecal samples. In these rat studies, the miniaturized PRIM device had 78% sensitivity and 72% specificity for detection of colitis (Figure 5E). These results are highly promising and, with further optimization, there is potential for the device to be tuned to approach the sensitivity and specificity of the current fecal calprotectin stool tests (89% sensitivity and 79% specificity).^54^

We note that there are limitations with this proof of concept *in vivo* experiment that necessitate further in-depth investigation in future studies. One limitation is the use of a chemically induced acute model of colitis to test our device. DSS-induced colitis is one of the most commonly used IBD models due to its simplicity, low cost, and control over colitis induction.^55–57^ It is recognized to induce neutrophil-mediated inflammation with similar pathophysiology to inflammation flare-ups in ulcerative colitis.^57^ However, this model may not represent the intermediate levels of chronic inflammation that would be important to detect in human patients between flare-ups. In future studies, it will be important to test the PRIM device in a chronic IBD model, such as a spontaneous IBD model (e.g., interleukin [IL]-2^− /−^ ) to determine the inflammation threshold it is capable of detecting and further tune it to detect inflammation levels relevant for managing human IBD. Another limitation is that the miniaturized device components used in rat experiments were manually manufactured and assembled, which could result in variances that affect the ROS threshold necessary for dye release. Moreover, the characteristics of the ROS-responsive polymer could be further optimized based on the type and amount of plasticizer used—while the miniaturized rat devices were manufactured with ROS-responsive dextran containing unreacted phenylboronic ester units, purification of the polymer followed by addition of plasticizers, such as ATBC, could provide more precise control in tuning the polymer’s mechanical properties and H_2_O_2_ sensitivity. The 3D-printed 00-sized devices paired with the ATBC-plasticized polymer offer more consistency over device performance, as demonstrated by *in vitro* release studies (Figures 4D and 4E). Furthermore, the larger size of the 3D-printed devices also provides the opportunity to optimize the cap design and the placement of ROS-responsive polymer adhesive to modulate the PRIM device’s sensitivity to H_2_O_2_. However, insights on the sensitivity and specificity of the 00-sized PRIM device would require *in vivo* studies using large-animal models.

While out of the scope of the current study, future large-animal studies would be necessary prior to translation of PRIM to humans. In these studies, comparison of the 00-sized PRIM device directly against a standard clinical measurement of intestinal inflammation (i.e., fecal calprotectin) would provide greater insight into the association of dye release from PRIM and inflammation levels. Our current study design uses stool consistency scoring to test that inflammation is induced after DSS administration and each rat acts as its own control where miniaturized PRIM devices are administered prior to colitis induction and after colitis induction. To control for the effect of time and change in inflammation state over time, future studies should follow animals longer term through the recovery phase from acute colitis using fecal calprotectin testing to compare how PRIM performs during and after the process of mucosal healing. Ideally, PRIM devices will be able to detect mild and moderate levels of inflammation in addition to severe inflammation. If our current PRIM design is not sufficiently sensitive to mild or moderate levels of inflammation in large-animal testing, there are a few options to tune the device. One approach is to tune the amount of polymer used to keep the device sealed. This can be done by applying a thinner layer of polymer to the device or by modifying the lid design to decrease the width of the notch that the polymer is added to. Another way to tune the device release is to change the design of the capsule lid. We have performed very preliminary studies with a mesh lid design that shows more rapid dye release (Figure S17). The type and amount of plasticizer may also be varied to enhance sensitivity. Further development of modified PRIM designs would allow us to achieve higher sensitivity. GI transit time will also be an important consideration when optimizing the device release threshold. Humans with IBD typically have slower GI transit time than healthy people, but more variability is observed. It will be critical to refine PRIM so that it is stable in the healthy GI tract in maximum transit times (>56 h) and is able to release its payload in inflamed conditions over relevant transit times.^52^ Diarrhea in people with IBD can cause very rapid transit times of around 9 h. It may be necessary to consider as PRIM is tested for clinical use whether guidelines are required that, if diarrhea occurs in a short window (e.g., 12 h) after administering PRIM, a new capsule should be taken to prevent a false-negative reading from device clearance. Future studies that look at the long-term stability of the plasticizer effect on the polymer to understand whether device performance is affected by storage time will also be important in optimizing the device.

In conclusion, this study introduces the PRIM device, an innovative ingestible biosensor designed for minimally invasive and accessible monitoring of intestinal inflammation in patients with IBD. The PRIM device utilizes an ROS-responsive polymer adhesive that selectively degrades in the presence of elevated H_2_O_2_, allowing for targeted release of dye cargo. Our results demonstrate that the device remains stable in the absence of ROS and only activates under elevated ROS conditions *in vitro*, providing a reliable visual indicator of intestinal inflammation. In rat models of colitis, the miniaturized PRIM device successfully detected inflammation with 78% sensitivity and 72% specificity. The larger, 3D-printed PRIM devices offer greater consistency in performance and provide opportunities for design adjustments to enhance sensitivity and control the release mechanism. Further optimization and testing of the 3D-printed PRIM device in large animals should enable the sensitivity and specificity of PRIM to approach that of existing fecal calprotectin tests. The PRIM device presents a promising, cost-effective alternative for frequent at-home monitoring of intestinal inflammation. By eliminating the need for stool handling and laboratory analysis, the PRIM test can potentially improve patient adherence and enable more consistent tracking of disease activity. Moreover, PRIM devices are predicted to be inexpensive to manufacture at scale and, since laboratory analysis of samples is unnecessary, it could further improve access to inflammation monitoring, particularly in low-resource settings. Widespread use of the PRIM device as a routine biomarker test could enhance patient-provider communication and support personalized treatment strategies, ultimately contributing to better disease management and improved outcomes for IBD patients.

## METHODS

### Materials

All reagents were purchased from Sigma-Aldrich unless otherwise stated. Pinanediol, 4-(hydroxymethyl) phenylboronic acid, and carbonyldiimidazole (CDI) were purchased from Chem-Impex.

### Synthesis of phenylboronic ester (1) *(4-((3a*S,*4*S,*6*S,*7a*R*)-3a,5,5-trimethylhexahydro-4,6-methanobenzo[*d*][1,3,2]dioxaborol-2-yl)phenyl) methanol*

4-(hydroxymethyl) phenylboronic acid (15.2 g, 100 mmol), and pinanediol (17.0 g, 1 eq) were heated at 110°C until molten and then stirred together. After 1 h, the pressure was slowly reduced, and stirring continued for 24 h under vacuum and heat to remove water byproduct from the condensation reaction. After cooling, the viscous yellow syrup product was obtained in quantitative yield and used without further purification. ^1^H NMR (400 MHz, CDCl_3_) δ 7.86–7.78 (m, 2H), 7.41–7.34 (m, 2H), 4.72 (s, 2H), 4.45 (dd, *J* = 8.8, 1.9 Hz, 1H), 2.51–2.35 (m, 1H), 2.30–2.20 (m, 1H), 2.19–2.13 (m, 1H), 2.05–1.90 (m, 2H), 1.49 (s, 3H), 1.32 (s, 3H), 0.90 (s, 3H).

### Synthesis of CDI-activated phenylboronic ester (2) *4-((3a*S,*4*S,*6*S,*7a*R*)-3a,5,5-trimethylhexahydro-4,6-methanobenzo[*d*][1,3,2]dioxaborol-2-yl)benzyl 1*H*-imidazole-1-carboxylate*

Synthesis of the CDI-activated phenylboronic ester was adapted from Wang et al.^58^ Phenylboronic ester 1 (28.6 g, 100 mmol) and CDI (24.3 g, 150 mmol) were dissolved in DCM (150 mL) and stirred at room temperature for 3 h. The reaction mixture was washed with deionized water until the aqueous layer was colorless (3 × 50 mL). The organic layer was dried with anhydrous MgSO_4_, filtered, and concentrated under reduced pressure. The product crystallized upon standing to a yellow-beige solid (37.0 g, 97% yield). ^1^H NMR (400 MHz, CDCl_3_) δ 8.14 (t, *J* = 1.1 Hz, 1H), 7.90–7.83 (m, 2H), 7.47–7.40 (m, 3H), 7.06 (dd, *J* = 1.7, 0.8 Hz, 1H), 5.43 (s, 2H), 4.46 (dd, *J* = 8.7, 1.8 Hz, 1H), 2.49–2.33 (m, 1H), 2.29–2.18 (m, 1H), 2.22–2.12 (m, 1H), 2.02–1.90 (m, 2H), 1.49 (s, 3H), 1.32 (s, 3H), 1.20 (d, *J* = 10.9 Hz, 1H), 0.90 (s, 3H).

### Synthesis of ROS-responsive dextran (3)

Synthesis of ROS-responsive dextran was adapted from Manaster et al.^31^ Dextran (molecular weight [MW] 9,000–11,000 Da [7.5 g]), CDI-activated phenylboronic ester 2 (35.0 g, 92 mmol), and 4-dimethylaminopyridine (11.2 g, 92 mmol) were dissolved in DMSO (75 mL) and stirred at room temperature for 72 h. The reaction mixture was precipitated dropwise in 5% w/v aqueous KHSO_4_ (500 mL) and then centrifuged at 4,500 rpm for 15 min. The supernatant was discarded, and the precipitate was washed with 5% w/v KHSO_4_ and centrifuged twice more, then it was washed with deionized water and centrifuged (3 × 500 mL). The product was lyophilized to yield a white powder (27.9 g). For further purification, the crude product (3.0 g) was dissolved in ethyl acetate (30 mL) and washed with 1 M HCl (3 × 10 mL), deionized water (3 ×10 mL), and brine (30 mL). Centrifugation was needed to separate the aqueous and organic layers after each wash. The organic layer was dried using anhydrous MgSO_4_ and filtered. The polymer was precipitated dropwise into hexanes (120 mL) and centrifuged at 4,500 rpm for 5 min. The supernatant was discarded, and the precipitate was dried under vacuum to yield a beige solid (1.8 g).

### Polymer degradation assay

ROS-responsive dextran was placed in either water or an H_2_O_2_ solution (100 mM at preparation, but dilute H_2_O_2_ solutions undergo more rapid degradation and it is expected that the solution was <100 mM at time of use). The ROS-responsive dextran was monitored over 24 h, and photos were taken to document dissolution.

### Cap detachment force

Modified 00-sized test devices with two open ends were designed using Fusion 360 (Autodesk; Figure S8A; Data S2–S5). Other than the open ends, these devices were prepared the same as those used in release studies. Devices were then printed using a FormLabs Form 3+ 3D printer using FormLabs gray resin. Each device was fitted with a soft, 40A O-ring (McMaster-Carr). The lid was placed and glued onto the device body by applying 50 μL of ROS-responsive polymer plasticized with 44% w/w ATBC, prepared by dissolving 400 mg/mL of ROS-responsive dextran in 7:3 acetone:ATBC. Devices were left to air dry for at least 1 h. After drying, completed devices were placed into an adapter, washed, and leak tested by incubating in water over night in a shaking incubator at 37°C, 100 rpm. After successful incubation, devices were air dried before proceeding to experiments. The effects of 1, 10, and 50 mM H_2_O_2_ conditions on cap detachment force were tested at 24 and 48 h. Different concentration solutions of H_2_O_2_ were made in a pH 7 phosphate buffer, and 10 mL of solution was added to each well of a six-well plate. A custom adapter was designed, printed, and placed over each six-well plate. 00-sized test devices were placed into the adapters, with the lid-body junction of the device submerged in the H_2_O_2_ solution. The plates were incubated for 24 or 48 h. After incubation, the devices were air dried. Devices were placed into a custom test bench. A pushrod connected to a mechanical analyzer (MTest Quattro) was then used to push the lid off the device from the inside. The force-displacement curve was recorded, and maximal force to detach the cap was determined (Data S1).

### SEM imaging for ROS-responsive dextran

Samples of ROS-responsive dextran coatings (unplasticized, containing residual boronic ester, or plasticized with 44% w/w ATBC) were prepared. Stainless steel rods were dip coated (MTest Quattro) in the respective polymer solution (400 mg/mL in acetone). Devices were incubated in phosphate buffer, deionized water, or 50 mM H_2_O_2_ for 4 h or 24 h. After incubation, samples were frozen at − 80°C and lyophilized overnight. After lyophilization, samples were mounted and sputter coated with 6 nm of platinum (Leica ACE600). Samples were imaged using a scanning electron microscope (Hitachi S4700).

### Cytotoxicity

Adult human dermal fibroblasts (HDFs; Lifeline Cell Technology, FC0024) were cultured in Dulbecco’s modified Eagle’s medium (DMEM) supplemented with 10% fetal bovine serum (FBS) and 1% penicillin-streptomycin under standard conditions (37°C, 5% CO_2_). Cells at passage 7 were seeded at a density of 10,000 cells per well in 96-well plates (100 μL per well) and allowed to adhere for 24 h. The culture medium was then replaced with 100 μL of fresh medium containing either (1) ROS-responsive dextran polymer or (2) ROS-responsive dextran polymer blended with 44% w/w ATBC at final polymer concentrations of 0, 0.005, 0.01, 0.05, 0.1, 0.5, 1, and 10 mg/mL. Due to limited aqueous solubility, the 10-mg/mL formulation contained visible polymer particulates, which were also incubated with the cells. Following a 24-h incubation with polymer-containing medium, the medium was aspirated, and cells were gently washed with 100 μL of phosphate-buffered saline (PBS). Cell viability was then assessed using the CyQUANT MTT Cell Viability Assay (Thermo Fisher Scientific, Waltham, MA, USA) according to the manufacturer’s protocol. Absorbance was measured using a microplate reader, and data were normalized to untreated controls.

### Release assay

00-sized devices were designed using Fusion 360 (Autodesk) (Data S6 and S7). Devices were then printed using a FormLabs Form 3+ 3D printer using FormLabs gray V4 resin. Each device was fitted with a soft, 40A O-ring (McMaster-Carr). Each device body was loaded with an aqueous solution of erioglaucine disodium salt (600 mg/mL, 0.55 mL) (brilliant blue, Millipore Sigma). The lid was placed and glued onto the device body by pipetting 50 μL of 400 mg/mL ROS-responsive polymer in a 44% w/w ATBC solution. Devices were left to air dry for 1 h. After drying, completed devices were washed and leak tested by incubating in water over night in a shaking incubator at 37°C, 133 rpm. After successful incubation, devices were air dried before proceeding to experiments.

H_2_O_2_ solutions (0, 1, 10, and 50 mM) were prepared in a pH 7 phosphate buffer, and 10 mL of solution was added to each well of a six-well plate. Completed biosensor devices were added to the wells. The plates were incubated at 37°C, 100 rpm, and time points were taken at 8, 24, 30, 48, and 72 h. At each time point, 150 μL of the release medium (i.e., H_2_O_2_ buffer solution) was aliquoted, diluted 100–1,000×, and absorbance at 625 nm was determined. Concurrently, each device was moved into a new well filled with a fresh solution of H_2_O_2_ (10 mL) and incubated under the same conditions as before. The device was placed into new release medium at each time point. Device release was defined as 10% of total dye loaded released.

For miniaturized size-9 devices, a 14-gauge blunt-tipped steel needle (McMaster-Carr) was cut to 6-mm length. One end of the needle was sealed with ethylene-vinyl acetate (EVA) polymer (i.e., hot-glue stick) using a hot-glue gun. To make the cap, EVA polymer was deposited into blocks 2 mm thick. Using a 2-mm biopsy punch (Integra), cylinders of polymer were punched out. Each cylinder was then press fitted into a cap shape by pushing the 14-gauge needle into the EVA cylinder. Each device was filled with erioglaucine disodium salt (brilliant blue FCF, 5 ± 2 mg), leaving a 1-mm air gap at the top of the device body. The cap was then gently fitted on top of each device. Each device was then dip coated in a solution of 400 mg/mL of ROS-responsive dextran (containing residual phenylboronic ester) dissolved in acetone. After manufacturing, each device underwent a leak test, where devices were incubated overnight in water using a shaking incubator 37°C, 100 rpm (Beckman-Coulter). Devices were incubated in a 24-well plate with 2 mL of release medium (0, 0.5, 1, and 50 mM H_2_O_2_) at 37°C, 100 rpm, and time points were collected at 0.25, 0.5, 0.75, 1, 2, 3, 5, 6, 28, 54, and 78 h. At each time point, each device was moved into a new well filled with fresh release medium (2 mL).

### Stability tests

00-sized PRIM devices were manufactured as described above for release assays. Devices were incubated for 72 h at 37°C and 100 rpm in various release media (10 mL): (1) phosphate buffer pH 2, (2) pH 8, (3) PBS pH 7.4, (4) 50 mM H_2_O_2_ (pH 7.4), (5) 50 mM HOCl/NaOCl (pH 8), (6) 3% w/v mucin, (7) simulated gastric fluid (0.64% w/v gastric pepsin, 0.2% NaCl, pH 1.2), and (8) simulated chyme (i.e., applesauce). At each time point (2, 4, 6, 24, 48, and 72 h), devices were transferred to a fresh solution of release medium (10 mL). Time-point samples (100 μL) were collected, diluted 10–1,000×, and the absorbance measured at 625 nm. Device release was defined as 10% of total dye loaded released. Dye release was quantified using brilliant blue FCF standards (0, 3.13, 6.25, 12.5, 25, and 50 mg/mL in PBS). For mucin and simulated-chyme tests, absorbance measurements were not taken due to turbidity of the release medium—device release was determined visually, and no blue dye was observed.

For simulated-gastric-mixing tests, devices were incubated in an *ex vivo* porcine stomach filled with simulated gastric fluid (300–600 mL), and the sphincters were sealed by sutures. The stomach was mechanically shaken on a tilting shaker plate at 80 rpm and continuously prodded with rods to simulate peristaltic contractions for 2–3.5 h (Figure S18; Video S1). For simulated-intestinal-peristalsis tests, devices were incubated in sausage casings filled with applesauce and sealed with dialysis clips. A rolling pin was continuously rolled over the devices using a tilting shaker plate at 15 rpm over 72 h (Figure S19; Video S2). Device release was determined visually at the end of each test, and no blue dye was observed.

### Rat model of colitis

All animal studies were performed in accordance with the Guidelines for the Care and Use of Laboratory Animals and the Animal Welfare Act Regulations. All protocols were approved by the Brigham and Women’s Hospital Institutional Animal Care and Use Committee. Male Sprague-Dawley rats (8–10 weeks) were used for these studies. The device was miniaturized, as described above for release assays, to fit inside a size-9 capsule, commonly used for rat models of disease. After manufacturing, each device underwent a leak test, where devices were incubated overnight in water using a shaking incubator at 37°C, 100 rpm. Devices that passed the leak test were air dried and loaded into size-9 gelatin capsules for *in vivo* testing. The device was loaded into the gelatin capsule so that the cap of the device aligned with the cap of the gelatin capsule. The device-loaded capsules were administered to healthy Sprague-Dawley rats by oral gavage, where the device cap entered the esophagus of the rat first. After administration, the rats were monitored daily for 72 h, and fecal samples were collected. A strong magnet was used to retrieve the device from the feces. Following three administrations (days 0, 5, and 8) of devices in healthy rats, colitis was induced in the rats using DSS. DSS water (5.5% w/w) was administered to the rats for 7 days. Stool consistency was measured daily starting the first day of DSS administration (Figure S20). After 7 days, normal water was once again provided, and devices were immediately administered on days 21, 25, and 29. The rats were monitored daily for 72 h after each administration, and fecal samples were collected and visually inspected for blue dye. As before, the device was retrieved from feces using a strong magnet. Blue feces and device retrieval usually occurred at the same time point and were typically observed 1 or 2 days after the device administration (Figures S22and S23); however, four of the 36 devices were not recovered. Two rats with unrecovered devices had blue feces, and time to device clearance was assumed to be the day blue feces was observed. For the two rats with unrecovered devices and normal feces (one healthy, one with colitis), they were returned to social housing, but feces were monitored for two additional days. Since no blue feces was observed during this extra monitoring period, it was assumed that devices were missed in stool as it can be deeply embedded in fecal pellets and these two samples were recorded as having normal feces and device recovery.

### Statistics

All data are reported as mean ± standard deviation (SD) unless otherwise stated. Comparisons between cap disengaging force, tensile modulus, and fracture strain after incubation in different media were conducted using a one-way ANOVA with Dunnett *post hoc* comparison tests for multiple comparisons and with a t test (two-tailed) for single comparisons (GraphPad Prism 10) (Figures 3D–3F and S10). To identify rats with blue feces, visual observation was used. As each rat acted as its own control, we used repeated measures, and as each trial was a simple yes or no (blue feces or normal feces), we used logistic regression. To accommodate repeated measures appropriately, we implemented the logistic regression as generalized estimating equations within a generalized linear model with a logistic link function and a binomial error distribution in PROC GENMOD (SAS Version 9.4). See Supplemental Methods: SAS Code used for Figure 5D for data and code. The effect of colitis was tested with likelihood ratios. The effect of device transit time approximated by the number of days until the device was recovered or the appearance of blue dye in feces, whichever observation came first, was also included. For the two rats with formal feces whose devices were not recovered, a value of 3 was entered for device transit time. Least-squares means and standard error (SE) (i.e., mean probability corrected for rat) were calculated and plotted or reported in the text. To generate the receiver operator curve, the criteria for a yes was defined as have blue feces once, twice in a row, or three times in a row and the percentages were calculated based on the number of opportunities to observe these criteria in the experiment for each of the rats during the healthy and DSS periods (18 opportunities for once, 12 opportunities for twice in a row, and six opportunities for three in a row).

## RESOURCE AVAILABILITY

### Lead contact

Requests for further information and resources should be directed to and will be fulfilled by the lead contact, Caitlin Maikawa (caitlin.maikawa@mail.utoronto.ca).

### Materials availability

This study did not generate new unique reagents.

## Supplementary Material

Video 1

Video 2

Supplementary Information

Data S1

Data S3

Data S2

Data S5

Data S4

Data S6

Data S7

SUPPLEMENTAL INFORMATION

Supplemental information can be found online at https://doi.org/10.1016/j.device.2025.100865.

## Data Availability

The data and code reported in this paper are included in the supplemental information. Additional information will be shared by the lead contact upon request.

## Highlights

- An ingestible biosensor device triggered by reactive oxygen species
- Biosensor uses a colorimetric readout to eliminate the need for fecal sampling
- Readout is simple and cost-effective and does not require laboratory analysis

## THE BIGGER PICTURE

Monitoring intestinal inflammation is critical for the effective management of inflammatory bowel diseases (IBDs), which are chronic diseases affecting over 7 million people globally. Currently, intestinal inflammation is measured infrequently as monitoring requires fecal sample collection, which is undesirable due to patient discomfort when handling fecal samples. As a result, inflammation monitoring is hindered by poor patient compliance—only 50% of prescribed tests are returned for testing. A simple biosensor device that eliminates the need for fecal sample handling would improve patient-driven inflammation monitoring and effective disease management of IBD. Recent work in pill-based sensors for inflammation monitoring has harnessed electrochemical or genetic circuits for sensing changes in the gastrointestinal environment associated with inflammation. However, the complexity of these devices reduces the likelihood of clinical adoption and increases costs for patients. Here, we introduce an ingestible pill-based biosensor device, the pill for reactive oxygen species (ROS)-responsive inflammation monitoring (PRIM), that uses ROS-responsive polymers to trigger colored dye release in the presence of intestinal inflammation. The PRIM device’s simple dye release mechanism would eliminate fecal sample handling and analysis and is a promising candidate for a patient-friendly, cost-effective solution for intestinal inflammation monitoring.
