# Supplementary material for: A radically simple, ingestible colorimetric biosensor pill for cost-effective, non-invasive monitoring of intestinal inflammation
Source: Device. Author manuscript; Available in PMC 2026 Feb 11. (PMC12889894; doi:10.1016/j.device.2025.100865)
Supplement: Supplementary Information [file NIHMS2127061-supplement-Supplementary_Information.pdf]

DEVICE, Volume *3*

Supplemental information

A radically simple, ingestible colorimetric biosensor pill for cost-effective, non-invasive monitoring of intestinal inflammation

Zile Zhuang, Lucia L. Huang, Bo Chan Seo, Subhashini Pandey, Jeffrey M. Karp, Yuhan Lee, and Caitlin L. Maikawa

Supporting Information

Table of Contents

Figure S1. Synthesis scheme for ROS-responsive dextran
Figure S2. NMR of phenylboronic ester
Figure S3. NMR of CDI-activated phenylboronic ester
Figure S4. NMR ROS-responsive dextran
Figure S5. NMR ROS-responsive dextran – purified
Figure S6. SEM images of ROS-responsive dextran coatings
Figure S7. CAD drawings of capsule
Figure S8. CAD drawings of push-off test set-up
Figure S9. Cap detachment force experimental set-up
Figure S10. Cap detachment force of PRIM device
Figure S11. Dye release from PRIM stability assays (individual replicates)
Figure S12. Dye release from PRIM stability assays PBS, H_2O_2, HOCl/NaOCl
Figure S13. Dye release from PRIM stability assays simulated GI fluids
Figure S14. Dye release from PRIM stability assays pH
Figure S15. PRIM release assay (individual replicates and images)
Figure S16. Polymer coating optimization
Figure S17. Alternative device designs for tunability
Figure S18. Experimental setup for simulated gastric mixing
Figure S19. Experimental setup for simulated intestinal peristalsis
Figure S20. Stool consistency scoring chart
Figure S21. Stool consistency score in rats
Figure S22. Rat feces images from healthy rats
Figure S23. Rat feces images from DSS colitis rats
Figure S24. Device transit time
Figure S25. Colour change in rat feces after oral administration of food dye

Table S1. ROS-responsive dextran P values adjusted for Dunnet multiple comparisons
Table S2. ROS-responsive dextran with ATBC P values adjusted for Dunnet multiple comparisons
Table S3. PRIM Device Cost Estimate
Table S4. Comparison of PRIM cost to alternatives for monitoring

Supplemental Methods: SAS Code used for Figure 5D

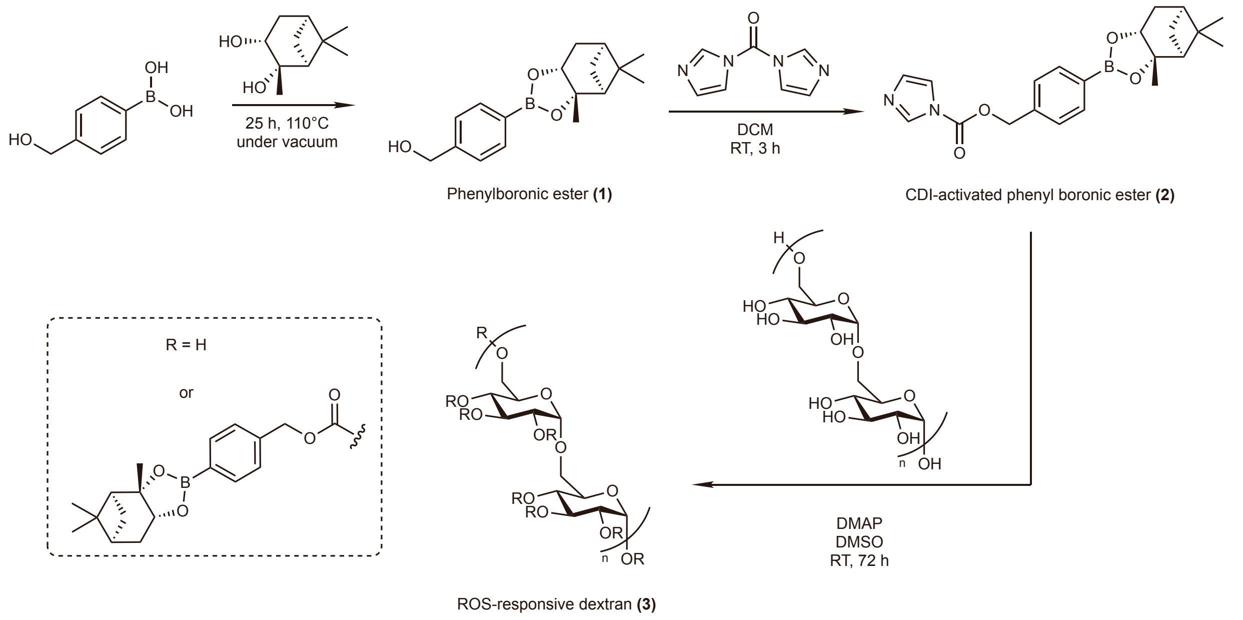

Figure S1. Synthesis scheme for ROS-responsive dextran.

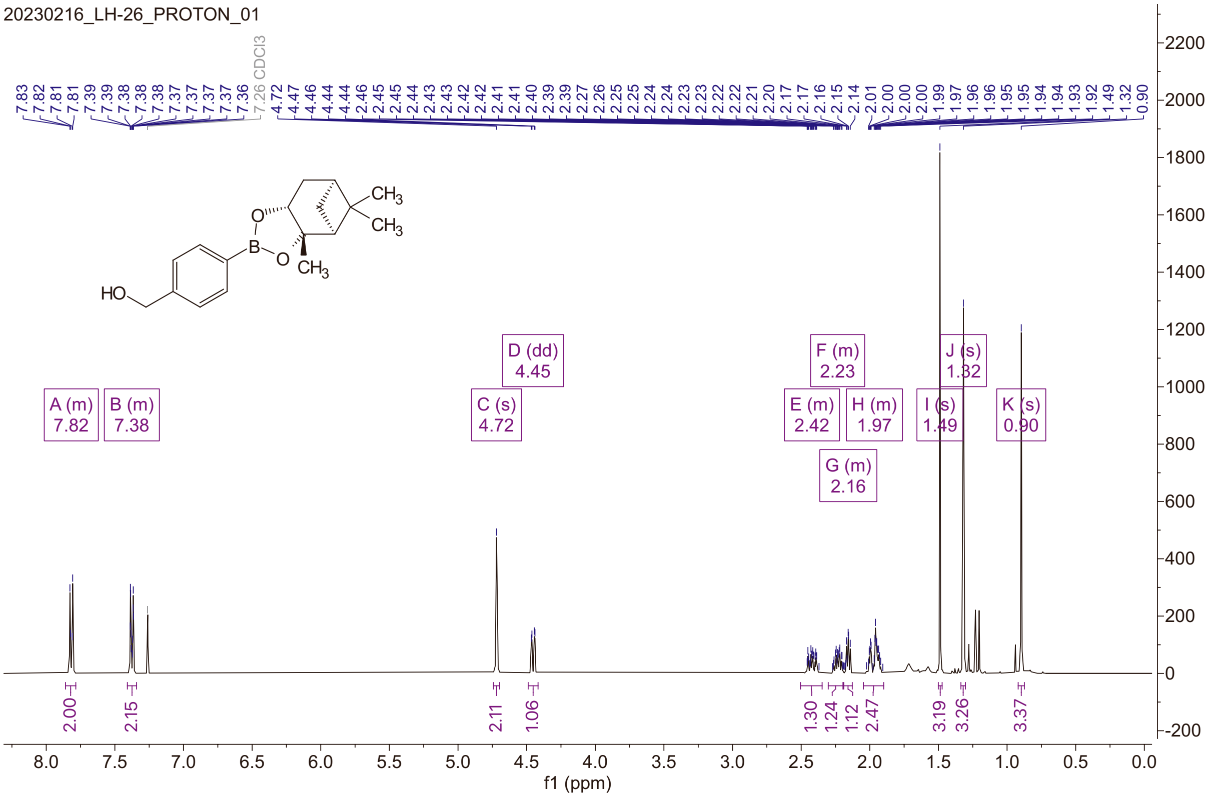

Figure S2. ^{1}H NMR ($CDCl_3$, 400 MHz) of phenylboronic ester

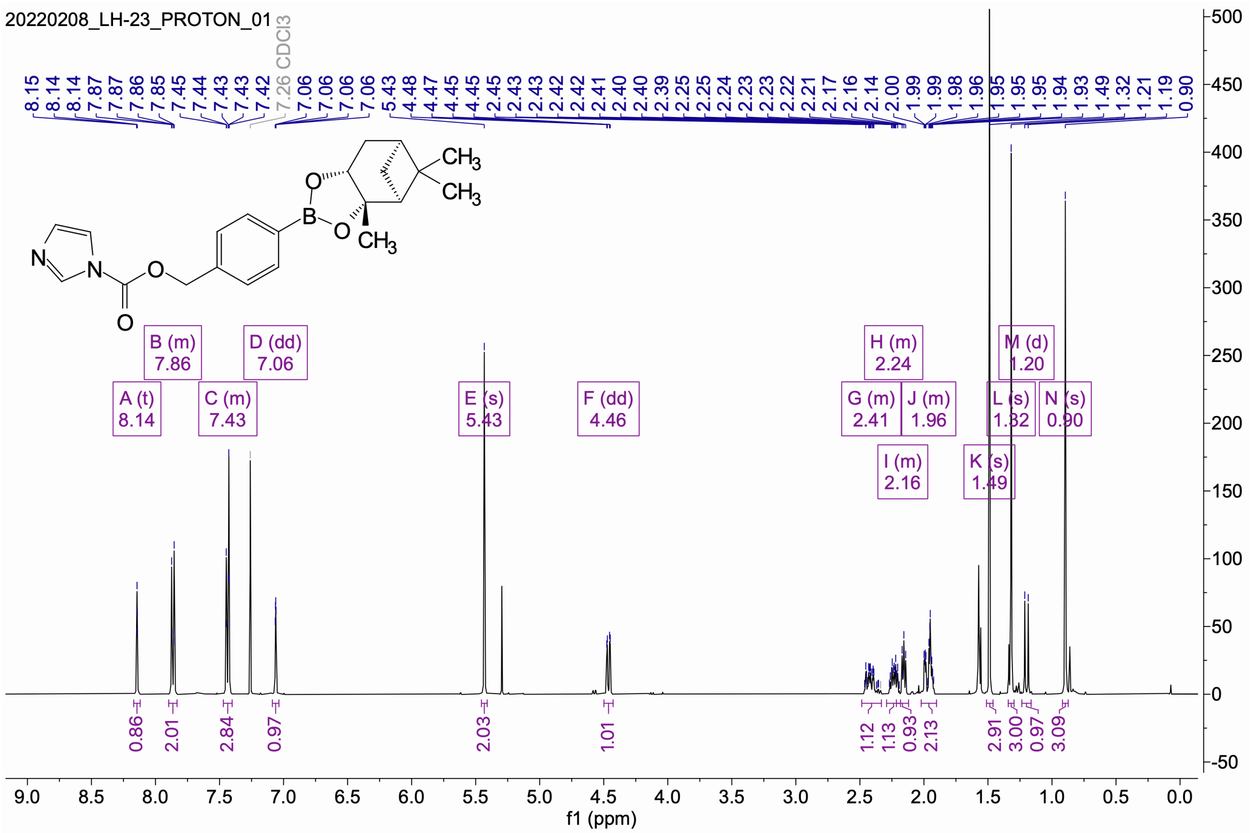

Figure S3. ^{1}H NMR ($CDCl_3$, 400 MHz) of CDI-activated phenylboronic ester

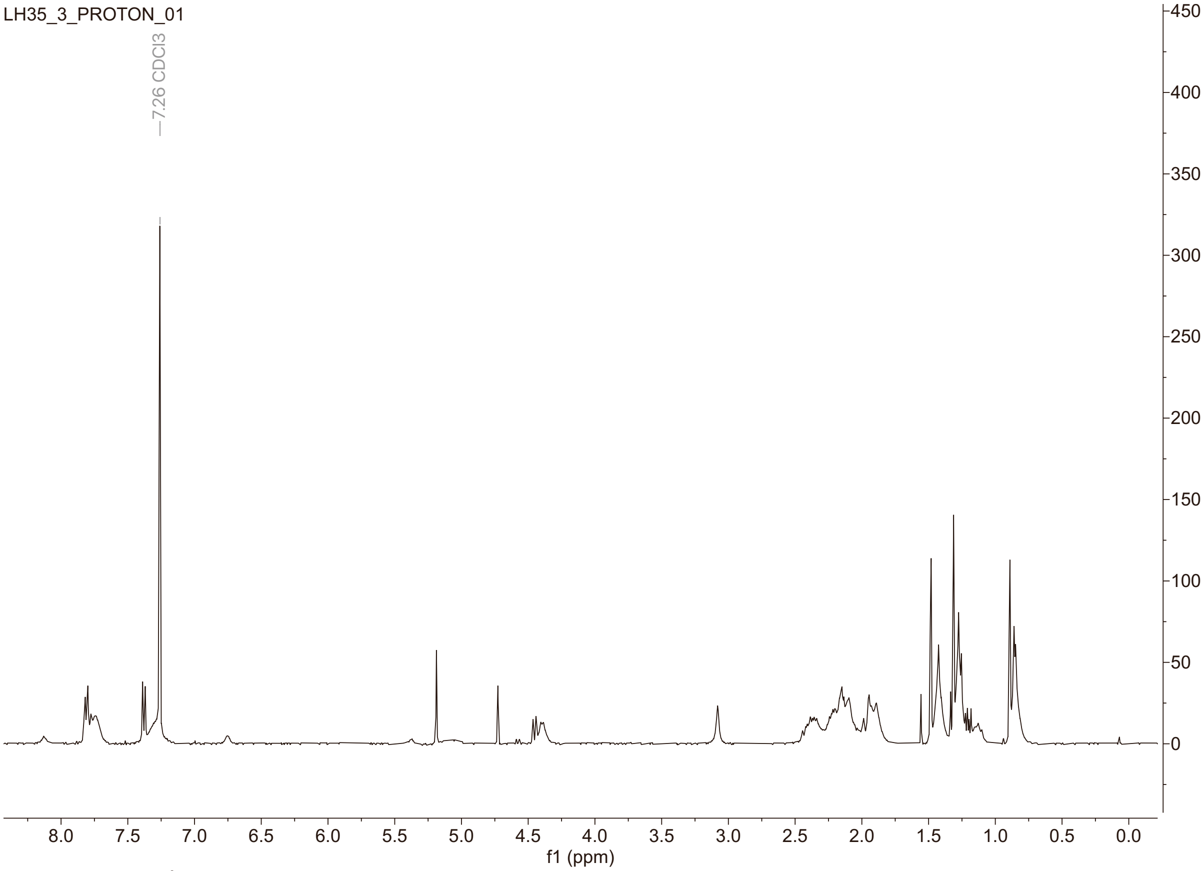

Figure S4. ^{1}H NMR ($CDCl_3$, 400 MHz) ROS-responsive dextran – containing unreacted phenylboronic ester.

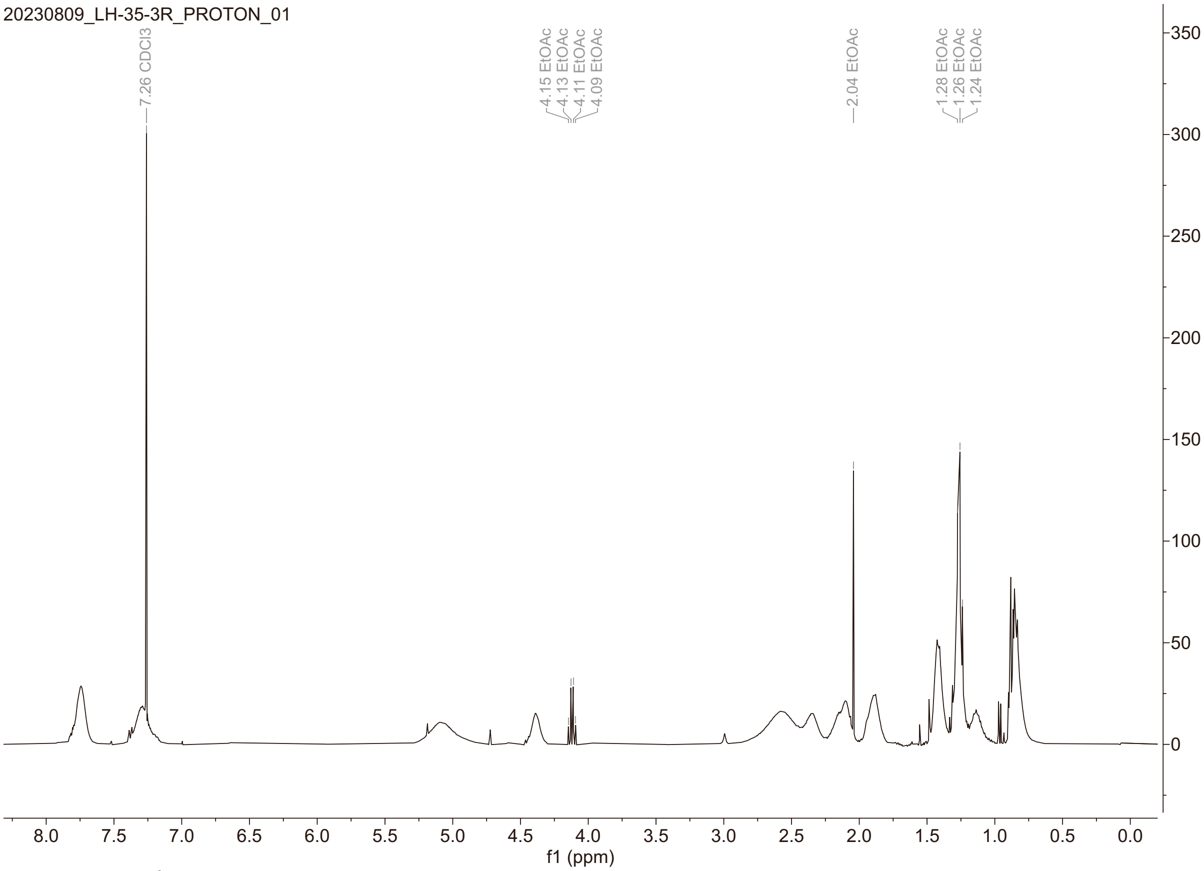

Figure S5. ^{1}H NMR ($CDCl_3$, 400 MHz) ROS-responsive dextran – purified.

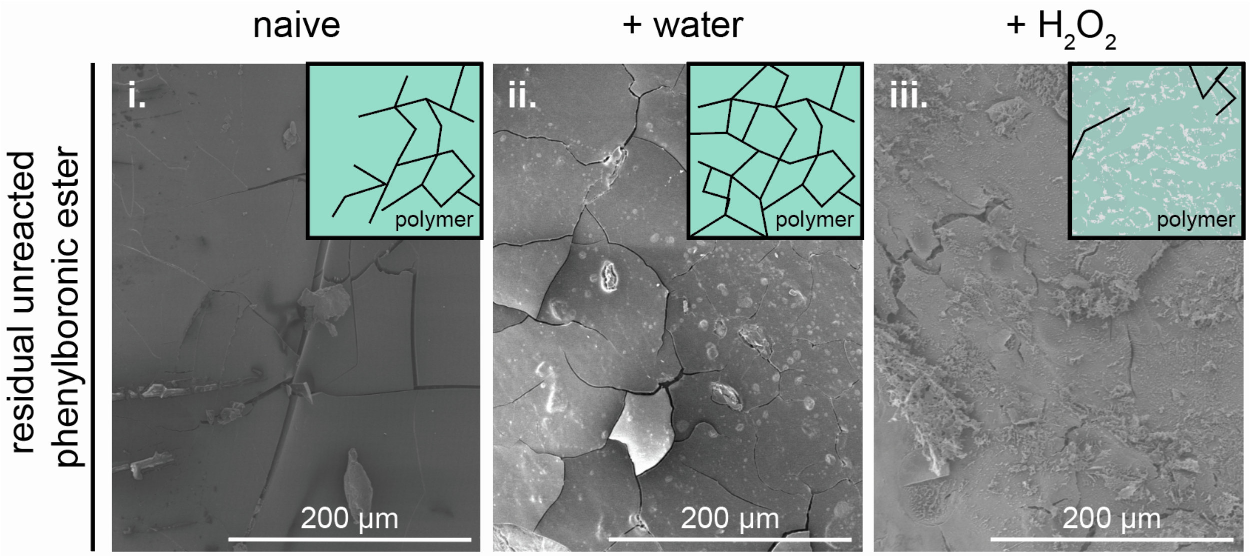

Figure S6. Scanning electron microscopy (SEM) images of ROS-responsive polymer. Coatings made with ROS-responsive dextran containing residual phenylboronic ester. Samples were (i) freshly prepared, (ii) exposed to water for 4 hours, (iii) exposed to 50 mM H_2O_2 for 4 hours prior to imaging.

Table S1. ROS-responsive dextran P values adjusted for Dunnet multiple comparisons

Comparison (mg/mL)	Adjusted P value
0 vs. 10	<0.0001
0 vs. 1	0.0467
0 vs. 0.5	0.0942
0 vs. 0.1	0.4791
0 vs. 0.05	0.3902
0 vs. 0.01	0.9254
0 vs. 0.005	0.9725

Table S2. ROS-responsive dextran with ATBC P values adjusted for Dunnet multiple comparisons

Comparison (mg/mL)	Adjusted P Value
0 vs. 10	<0.0001
0 vs. 1	0.0002
0 vs. 0.5	0.0050
0 vs. 0.1	0.0276
0 vs. 0.05	0.0419
0 vs. 0.01	0.3006
0 vs. 0.005	0.4997

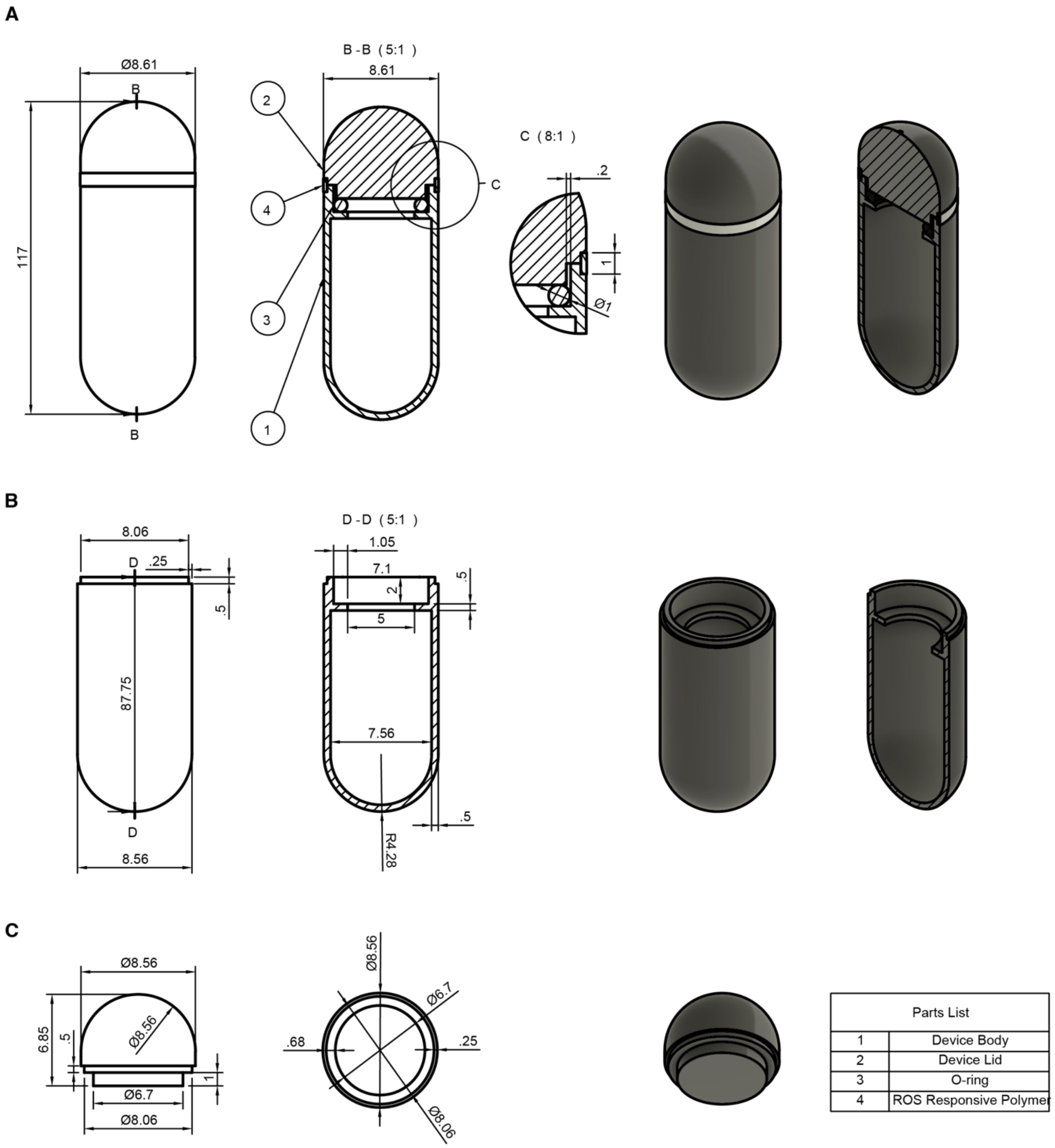

Figure S7. CAD drawings of capsule. CAD drawings of the capsule body and lid. **(A)** full capsule, **(B)** capsule body, **(C)** capsule lid.

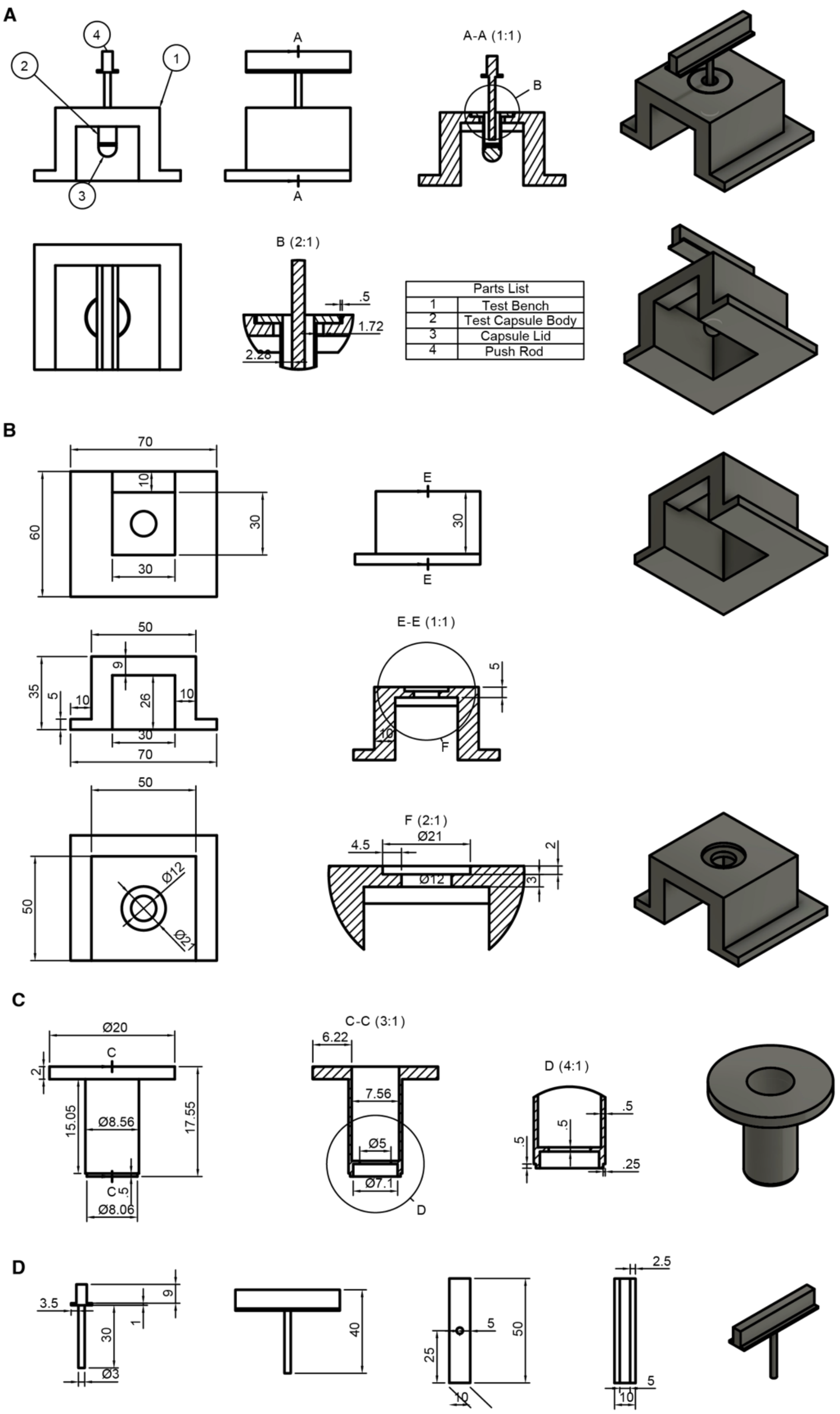

Figure S8. CAD drawings of push-off test set-up. CAD drawings of the 3D printed components for the cap detachment test including **(A)** full set-up, **(B)** test bench, **(C)** test capsule body, **(D)** push rod.

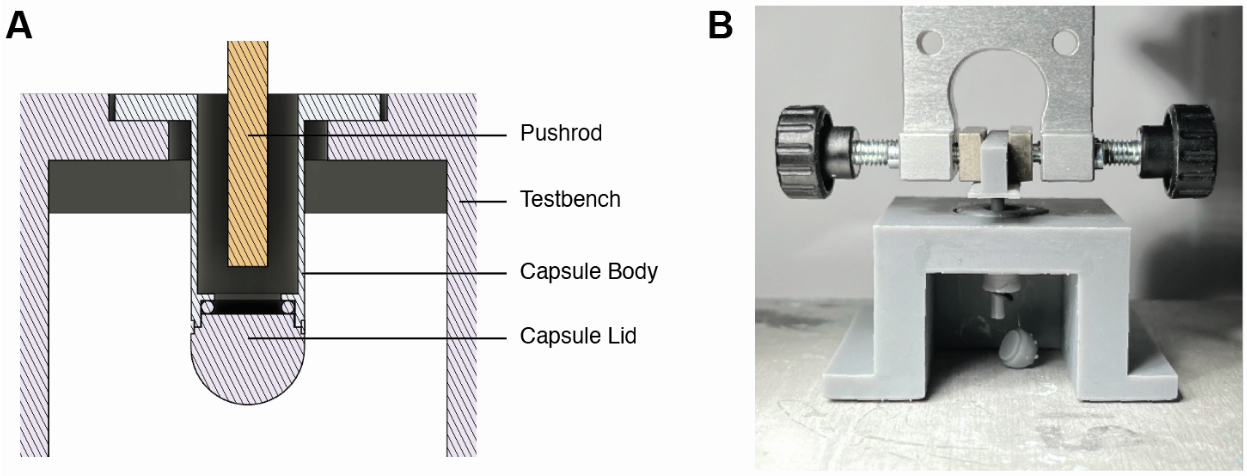

Figure S9. Cap detachment force experimental set-up. (A) Schematic of set-up for measuring cap disengaging force. **(B)** A mechanical analyzer (Admet) was used to record force displacement measurements. The modified PRIM devices were set up as pictured for the experiment.

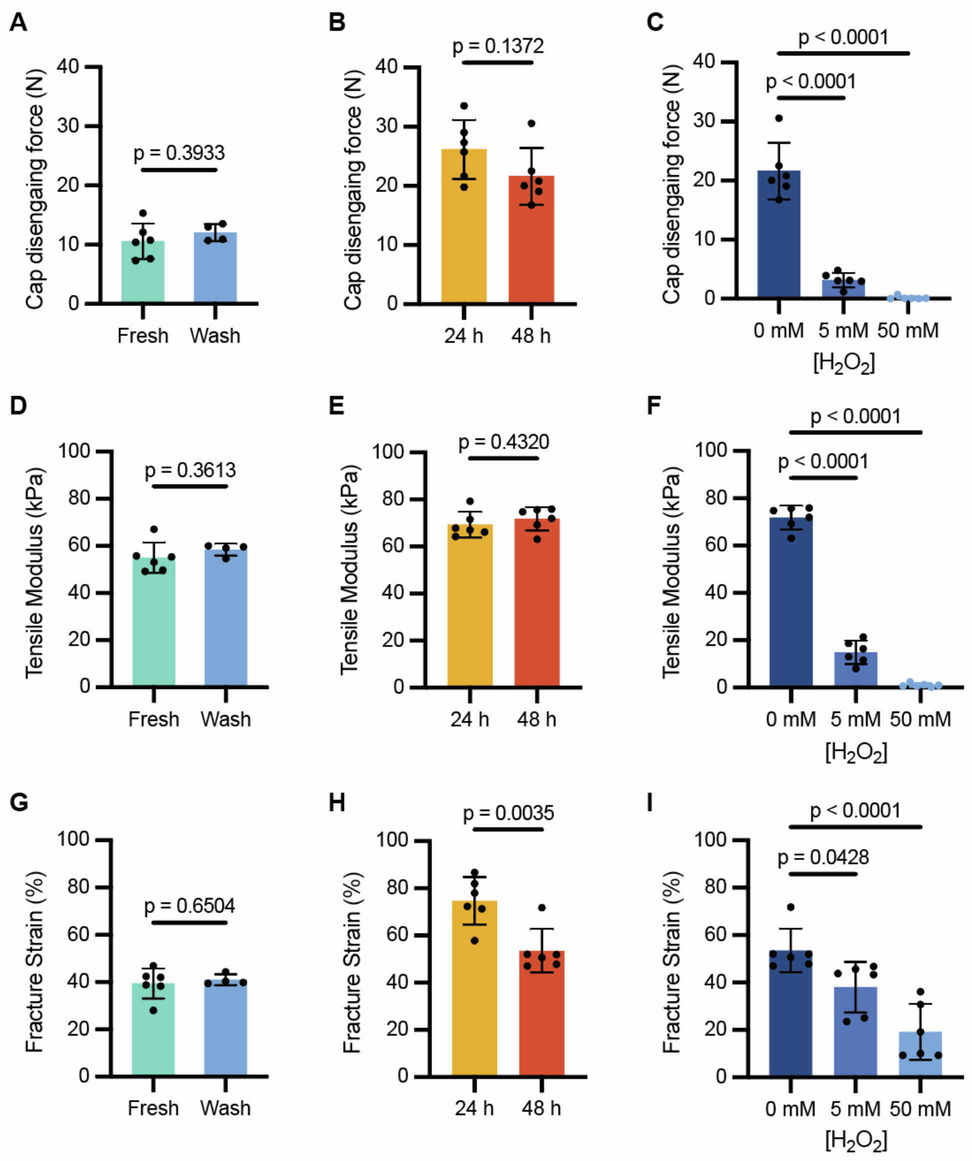

Figure S10. Cap detachment force of PRIM device. (A) Cap detachment force for fresh devices and for devices after an overnight wash in water (used to remove any dye from the outside of the device and test for leaking before further testing). **(B)** After washing in water and air-drying, devices were then incubated in phosphate buffer (pH 7) for 24 or 48 hours before testing. **(C)** Devices tested after incubation for 48 hours in 0 mM (i.e phosphate buffer), 5 mM, and 50 mM H_2O_2 solution. **(D-F)** Tensile modulus of polymer adhesive **(D)** for fresh devices and for devices after an overnight water in water, (E**)** for devices incubated in phosphate buffer (pH 7) for 24 or 48 hours, and **(F)** for devices incubated for 48 hours in 0 mM (i.e phosphate buffer), 5 mM, and 50 mM H_2O_2 solution. **(G-I)** Percent strain at fracture **(G)** for fresh devices and for devices after an overnight water in water, **(H)** for devices incubated in phosphate buffer (pH 7) for 24 or 48 hours, and **(I)** for devices incubated for 48 hours in 0 mM (i.e phosphate buffer), 5 mM, and 50 mM H_2O_2 solution. **(A, B, D, E, G, H)** Statistical significance was determined using a t-test (two-tailed). **(C, F, I)** Statistical significance was determined by one-way ANOVA. Dunnett post-hoc tests were applied for multiple comparisons to the 0 mM control. Data are represented as mean ± standard deviation.

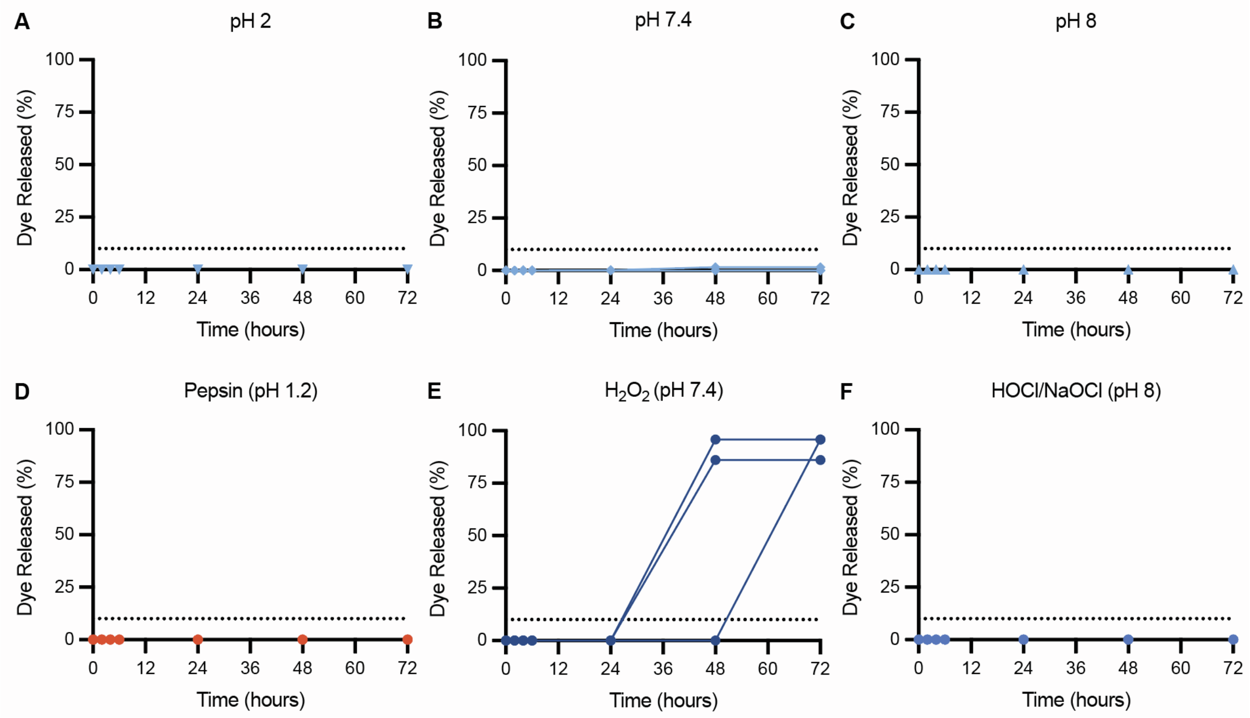

Figure S11. Dye release from PRIM stability assays (individual replicates and images). In vitro stability tests of PRIM device in **(A)** acidic conditions expected in the gastrointestinal tract (pH 2), **(B)** neutral conditions expected in the lower small intestine and colon (pH 7.4), **(C)** basic conditions that are present in short duration in the small intestine (pH 8), **(D)** digestive enzyme pepsin (pH 1.2), **(E)** ROS species (H_2O_2) **(F)** alternative ROS species (HOCl/NaOCl).

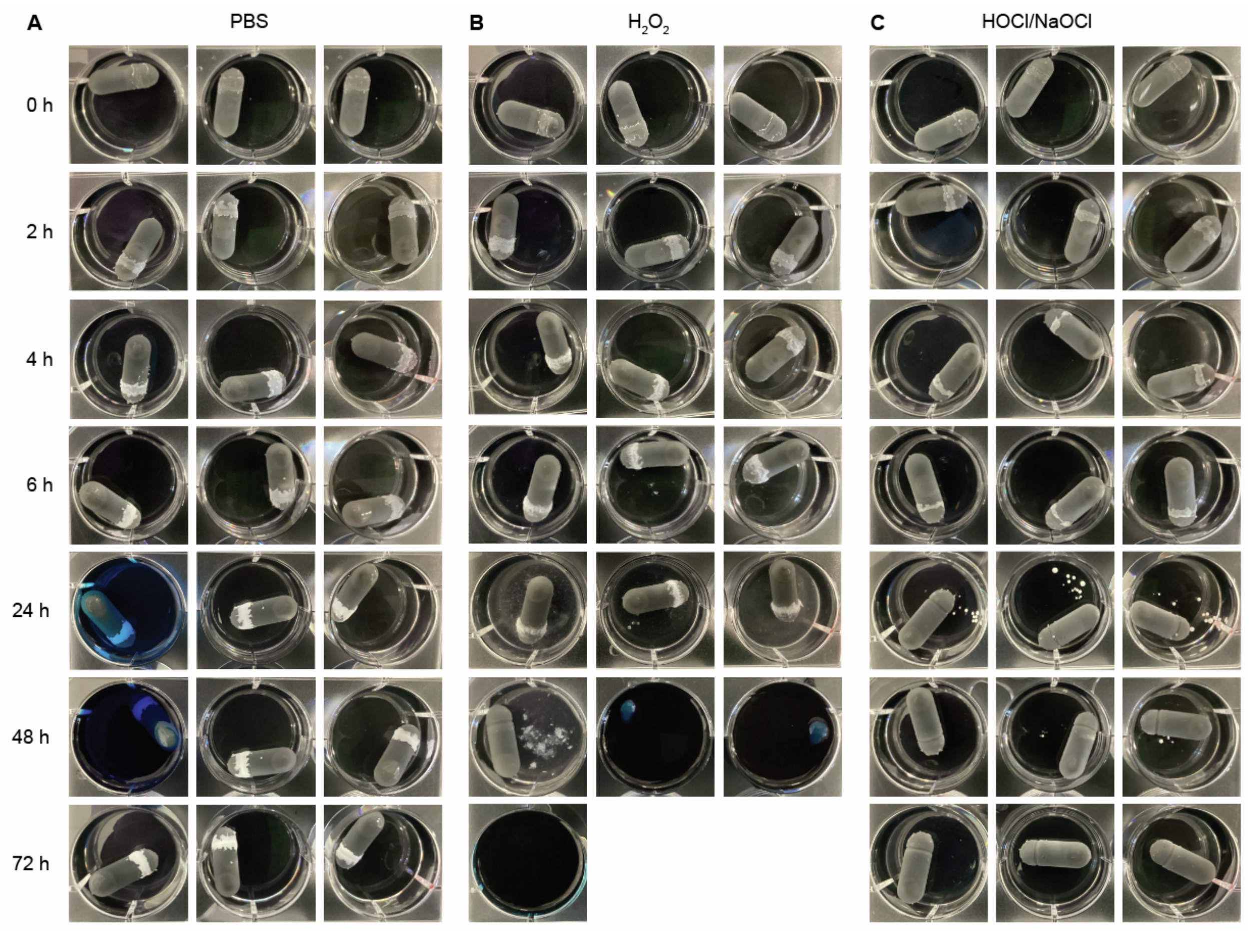

Figure S12. Dye release from PRIM stability assays PBS, H_2O_2, HOCl/NaOCl (individual replicates and images). Images of PRIM devices incubated in **(A)** phosphate-buffered saline (PBS, pH 7.4), **(B)** 50 mM H_2O_2 (pH 7.4), and **(C)** 50 mM HOCl/NaOCl (pH 8) from 0-72 hours for *in vitro* stability tests.

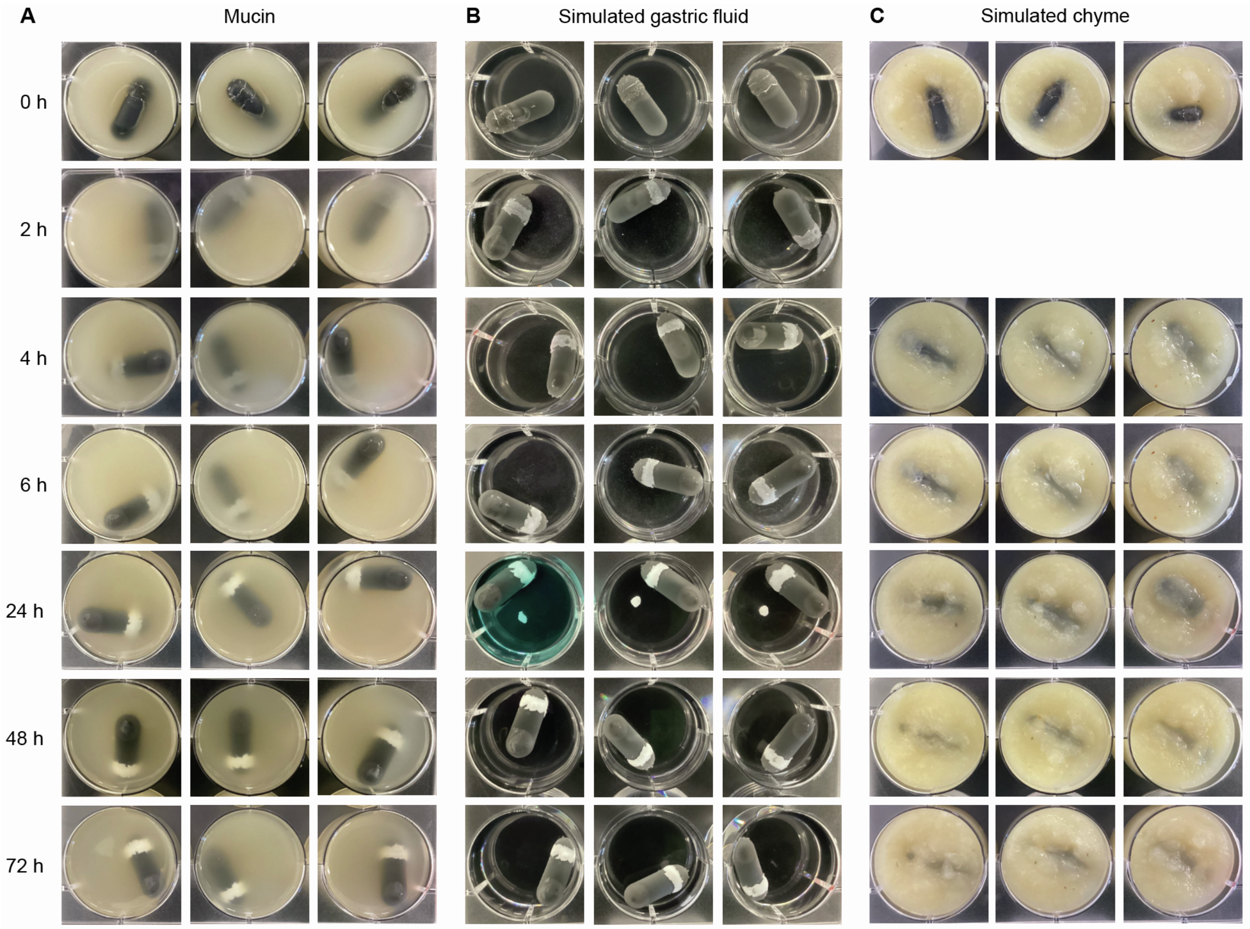

Figure S13. Dye release from PRIM stability assays simulated GI fluids (individual replicates and images). Images of PRIM devices incubated in **(A)** 3% w/v mucin **(B)** simulated gastric fluid (0.64% w/v pepsin, pH 1.2), and **(C)** simulated chyme (i.e. applesauce) from 0-72 hours for *in vitro* stability tests.

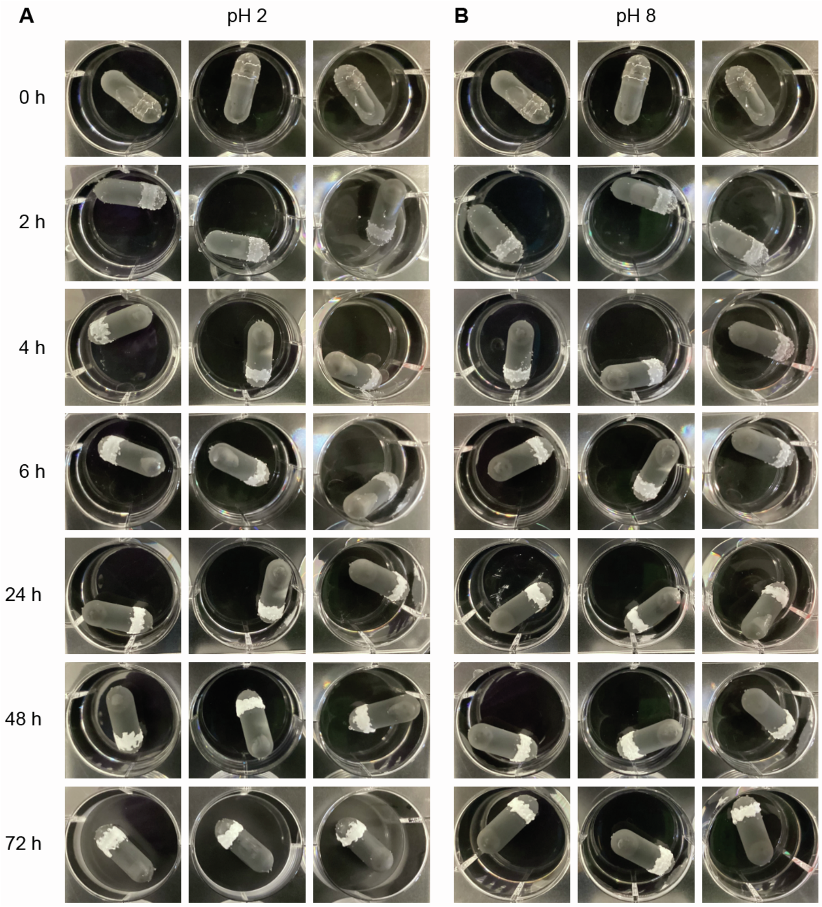

Figure S14. Dye release from PRIM stability assays pH (individual replicates and images). Images of PRIM devices incubated in **(A)** acidic pH (phosphate buffered saline, pH 2), and **(B)** alkaline pH (phosphate buffered saline, pH 8) from 0-72 hours for *in vitro* stability tests.

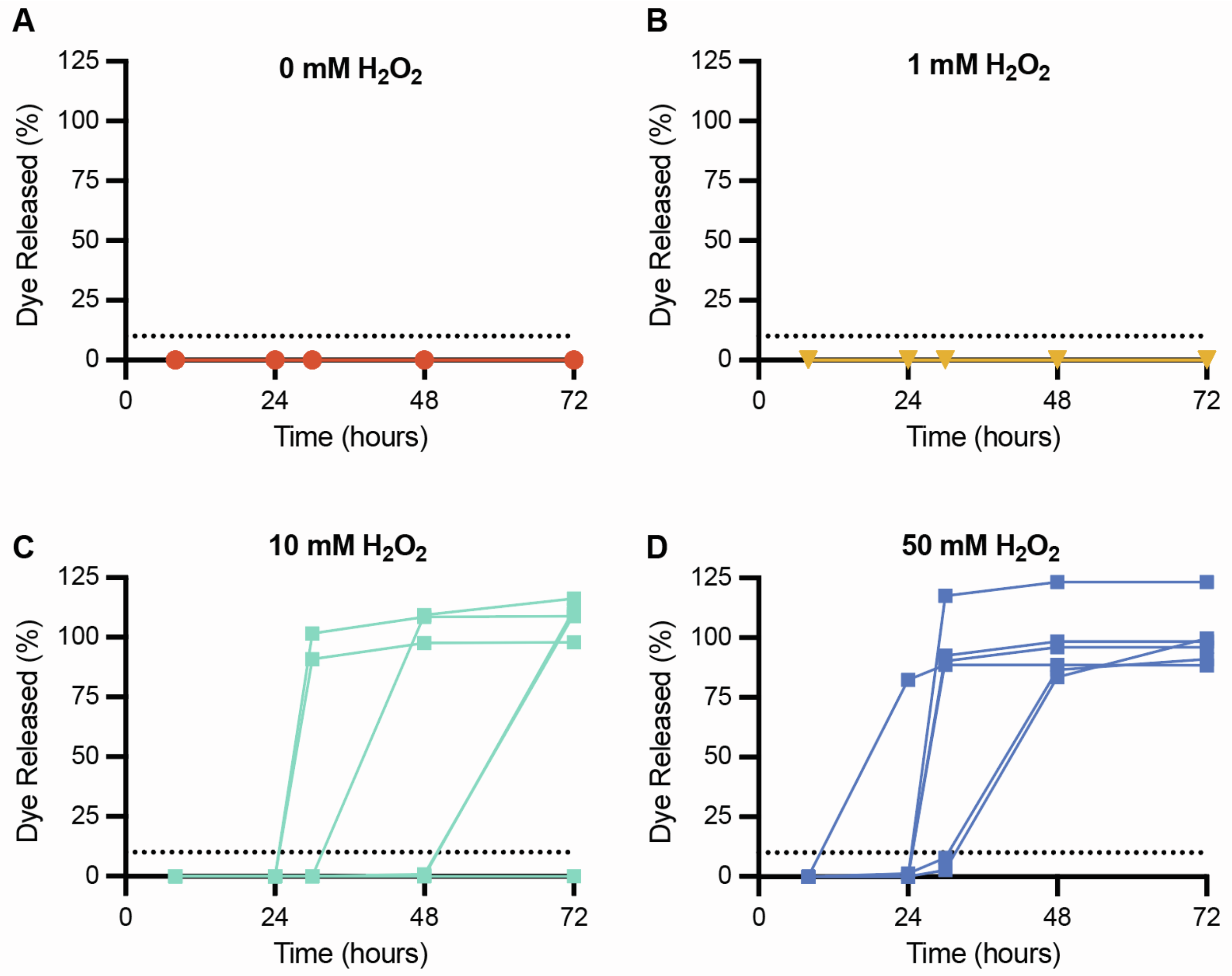

Figure S15. PRIM release assays (individual replicates). *In vitro* study of time to dye release from PRIM devices (n = 5 per group) in **(A)** 0 mM, **(B)** 1 mM, **(C)** 10 mM, and **(D)** 50 mM H_2O_2. Device release was achieved when >10 % of total loaded dye was released.

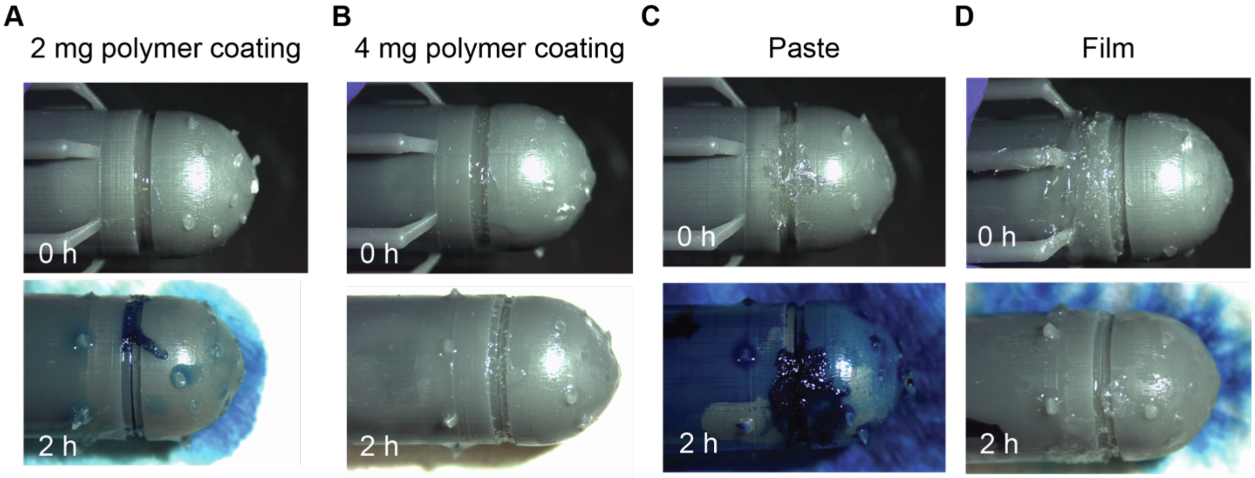

Figure S16. Polymer coating optimization. Devices with different applications of ROS-responsive polymer were incubated in 50 mM H_2O_2 for 2 hours to observe dye release. **(A, B)** Coatings were applied to devices by adding **(A)** 5 µL, (2 mg polymer) or **(B)** 10 µL (4 mg polymer) of a 400 mg/mL polymer in an acetone ATBC solution (30 % v/v ATBC). Dye released rapidly from the 5 µL, but it was difficult to tell if this was the result of insufficient polymer coating to hold the cap in place, or if it was ROS mediated polymer dissolution. Ultimately, a 50 µL (20 mg) coating was used for all experiments, but further tuning of polymer thickness has potential for tuning time to release or device sensitivity. **(C)** A polymer paste made by allowing the polymer/ATBC/acetone solution to begin to dry before application to the device to hold the cap to the device. **(D)** Polymer solution was dried as a film on a piece of PTFE liner, and then after drying, it was peeled from the PTFE liner and applied to the device to hold on the cap. **(C,D)** Dye released rapidly from these devices in 50 mM H_2O_2, however, the coating of the paste and film to the device did not result in an even coating and it was difficult to determine if dye release was a result of uneven coating application.

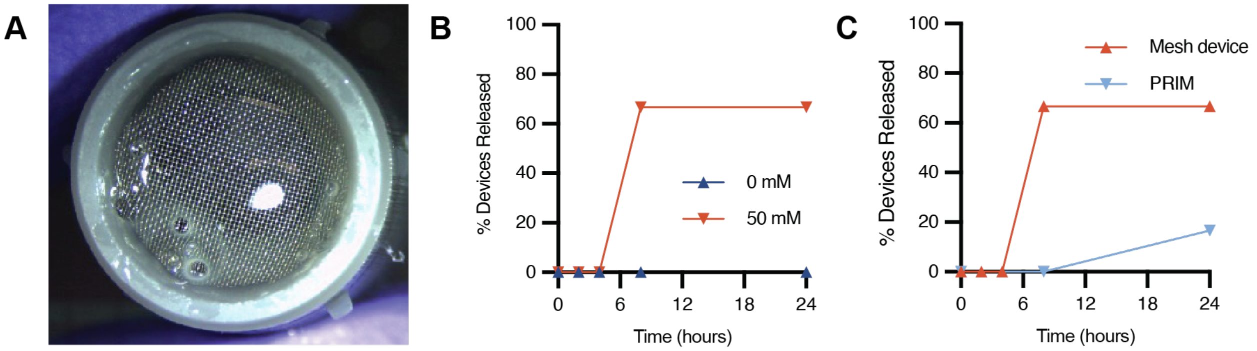

Figure S17 Alternative designs for tunability. (A) To tune the sensitivity of PRIM devices, changes to the device cap can be made. This could include changes to the notch depth or thickness to change the amount of polymer used to adhere the cap on, or it could be an alternate cap design. **(A)** A mesh lid design where a mesh is used to hold the dye in the capsule, and ROS-responsive dextran is used as a film coating the mesh lid. **(B)** Similar to the original design, the mesh lid device (n=3) releases dye at high H_2O_2 levels but does not release when in the absence of H_2O_2. **(C)** The mesh lid device shows greater sensitivity in comparison to PRIM and releases dye cargo more quickly in response to high levels of H_2O_2 (50 mM).

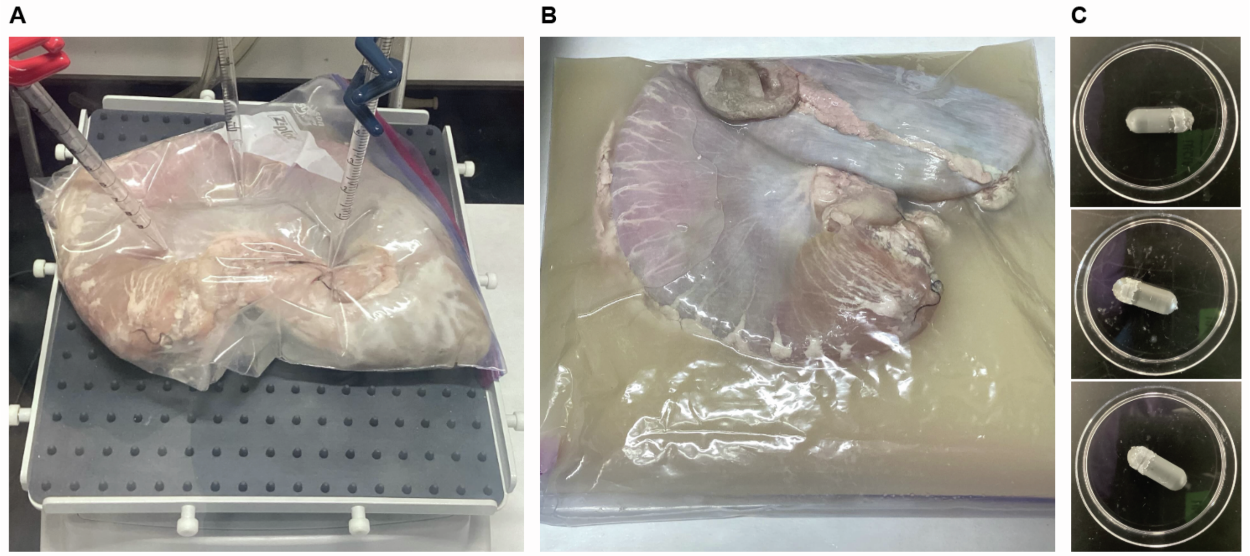

Figure S18. Experimental setup for simulated gastric mixing. (A) Image of *ex vivo* porcine stomach filled with simulated gastric fluid being mechanically agitated and prodded on a tilting shaker plate. **(B)** Representative image of stomach contents after 2-3.5 hours of simulated gastric mixing. **(C)** Images of PRIM devices recovered from stomach after simulated gastric mixing.

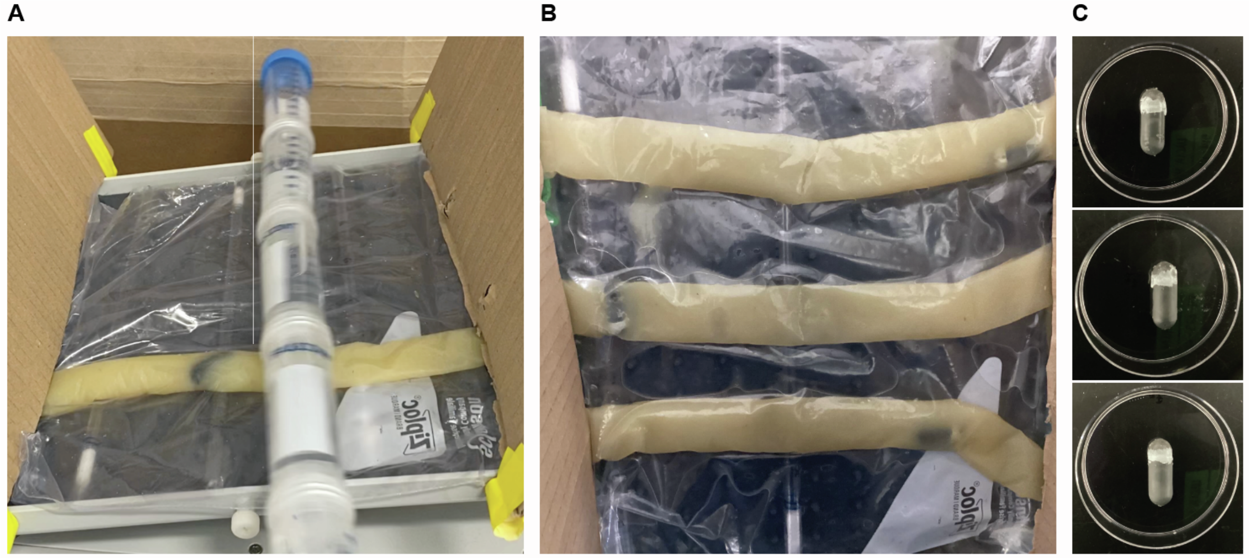

Figure S19. Experimental setup for simulated intestinal peristalsis. (A) Image of sausage casing filled with simulated chyme being mechanically squeezed by the motion of a rolling pin on a tilting shaker plate. **(B)** Image of simulated intestinal contents after 72 hours of simulated peristalsis. **(C)** Images of PRIM devices recovered after simulated peristalsis.

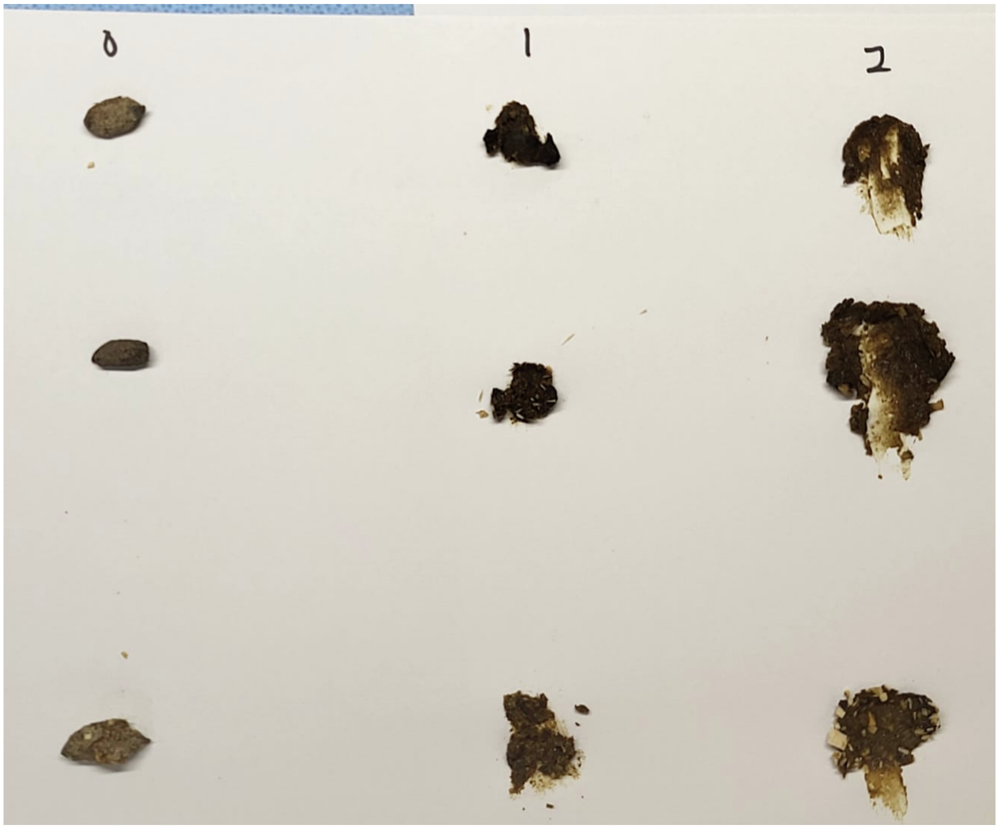

Figure S20. Stool consistency scoring chart. A stool smear test was used to score stool consistency as 0 = Normal, 1 = Soft, 2 = Very Soft, 3 = Diarrhea. Three replicates of each stool type is shown. (Not rats had liquid diarrhea and so this is not shown in our smear test chart).

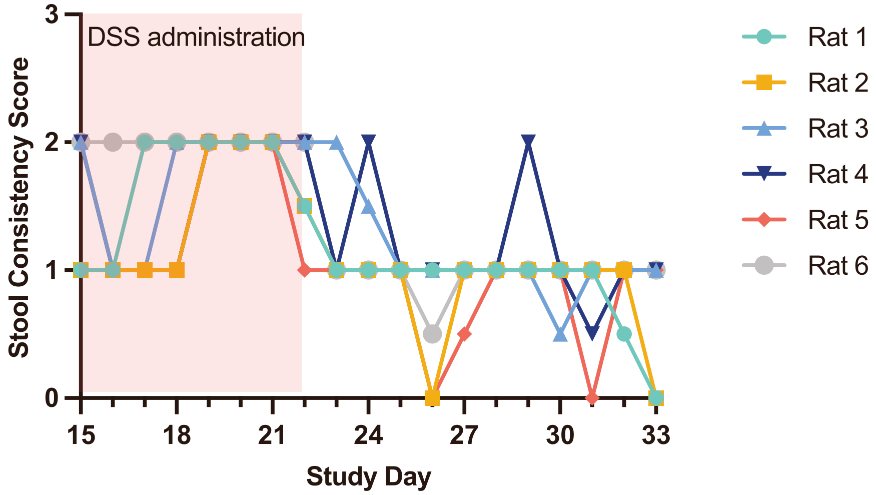

Figure S21. Stool consistency score in rats. Stool consistency scores for the rats during DSS administration and during the subsequent device testing period.

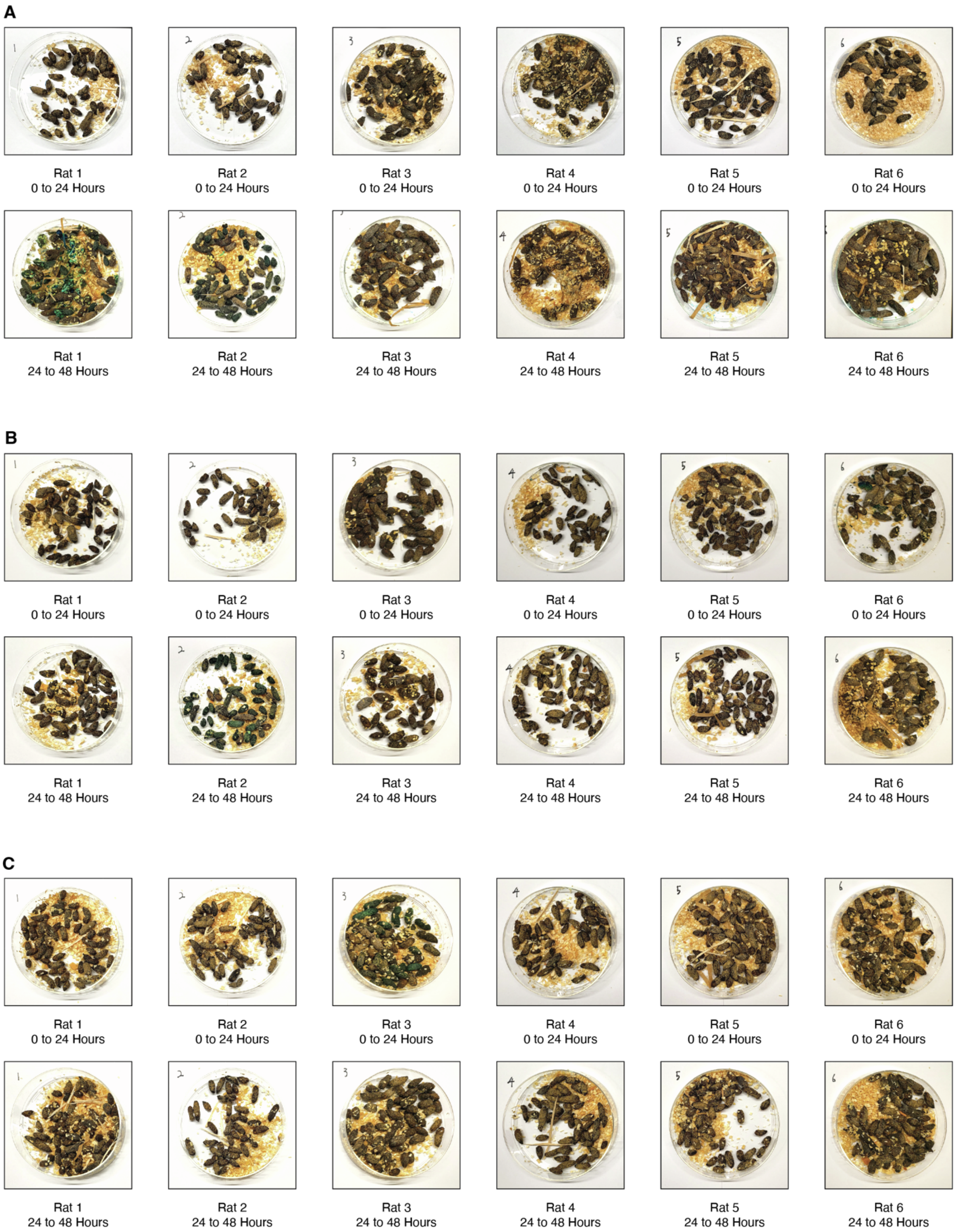

Figure S22. Rat feces images from healthy rats. Images show feces collected from rats for 0-24 hours and 24-48 hours following miniaturized PRIM (length = 7 mm) administration **(A)** Day 0 administration, **(B)** Day 5 administration, **(C)** Day 8 administration. Feces was checked for blue samples during this period and blue feces during either period. (Image brightness correction (+20%) was applied in Microsoft Word across all images to increase the ease of blue stool identification).

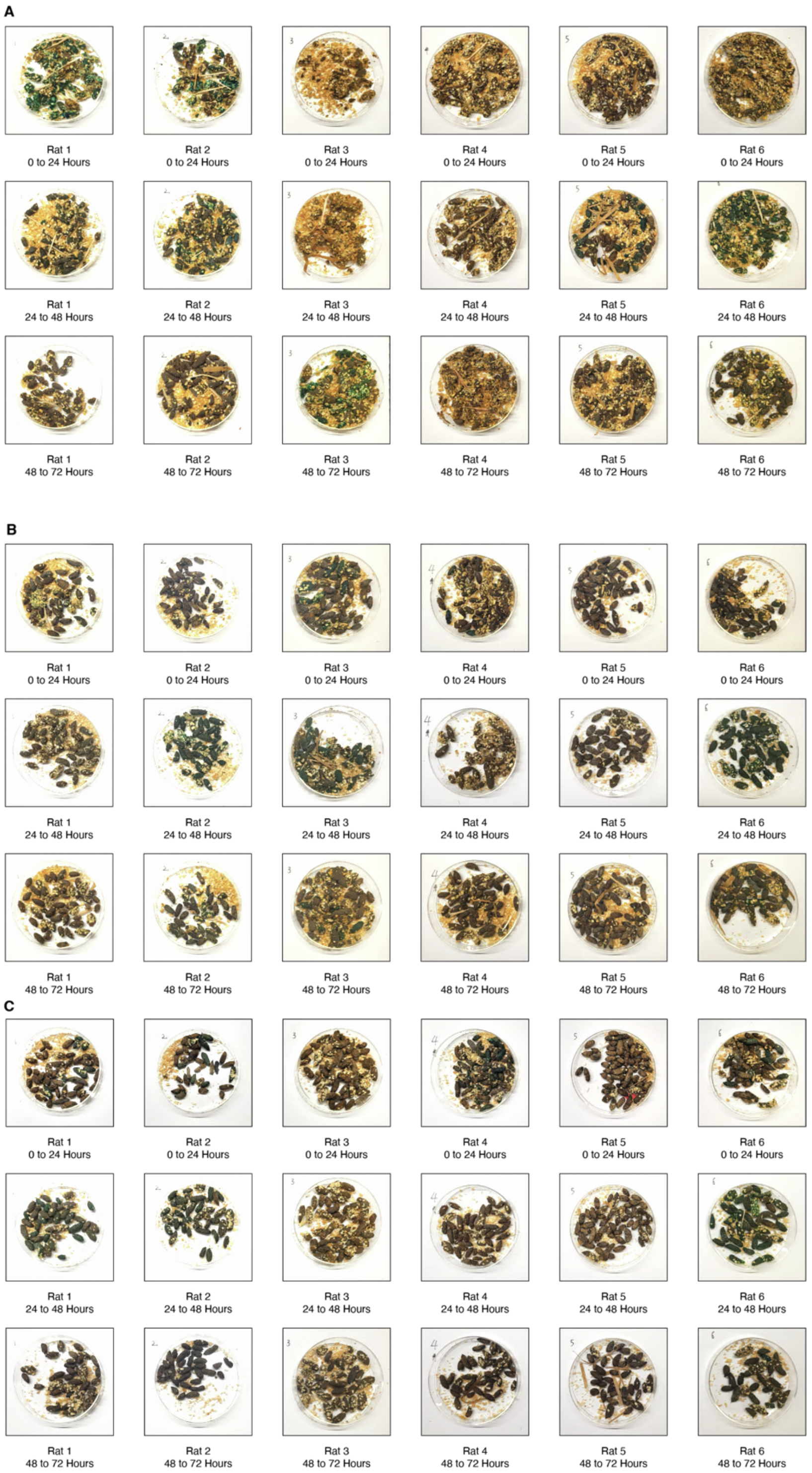

Figure S23. Rat feces images from colitis rats. Images show feces collected from rats for 0-24 hours, 24-48 hours, and 48-72 hours following miniaturized PRIM (length = 7 mm) administration: **(A)** Day 21 administration, **(B)** Day 25 administration. **(C)** Day 29 administration. (Image brightness correction (+20%) was applied in Microsoft Word across all images to increase the ease of blue stool identification).

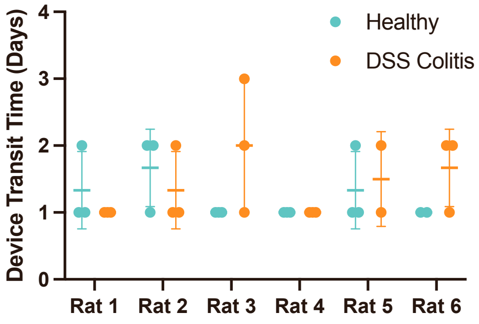

Figure S24. Device transit time. Device transit time was approximated by the number of days until the device was recovered or the appearance of blue dye in feces, whichever observation came first. Rat 5 from the colitis group, and Rat 6 from the Healthy group are each missing one data point as their devices were not recovered during the monitoring period. Data are represented as mean ± standard deviation.

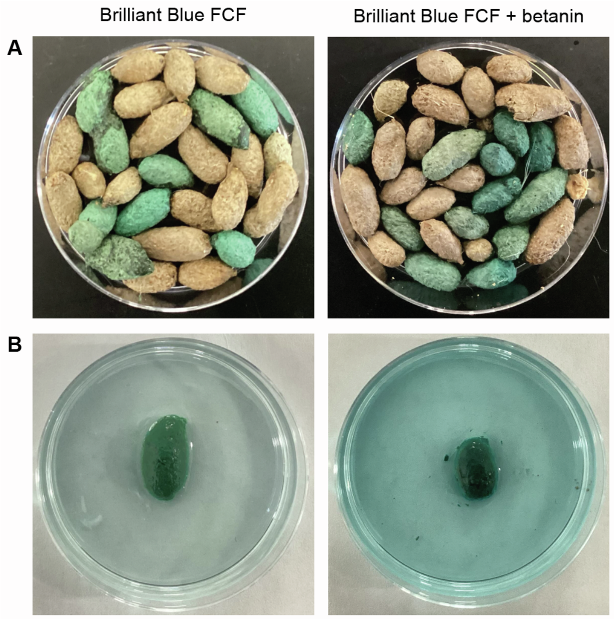

Figure S25. Colour change in rat feces after oral administration of food dye. (A) Image of rat feces collected 18 hours after administration of Brilliant Blue FCF (5 mg) (left), and co-administration with betanin (120 mg) (right). Both sample sets show visibly blue feces. **(B)** Image of blue feces in water (after 5 minutes). Surrounding water (e.g. toilet bowl) turns visibly blue.

Table S3. PRIM Device Cost Estimate

Material	Quantity (per device)	Bulk reagent cost	Cost
**ROS-responsive dextran polymer**	20 mg	$5/g	$0.10
**ATBC**	22 mg	~0.15/g[1]	<$0.01
**Brilliant Blue**	330 mg	~$0.50/g[2]	$0.17
**Device capsule**	1 capsule	$0.10/capsule (at scale injection molding)	$0.10
**Total**			$0.38

[1] Sigma Aldrich ($82 for 500mL ATBC)
[2] Ingredient Depot ($219 for 454 g of Brilliant Blue Food Dye)

Table S4. Comparison of PRIM cost to alternatives for monitoring

PRIM	Fecal calprotectin test	Wireless capsule endoscopy (ex. PillCam)	Estimate of other wireless capsule technology (ex. Smart Capsule)[60]
Cost is consists of device components.<br><br><u>Additional Requirements:</u><br>None. Visual readout requires no specialized personnel or tools.	Cost estimate from Medicare reimbursement[2]<br><br>Code: 83993<br>Year: 2025<br><br><u>Additional requirements:</u><br>Fecal sample collection by patients and then sample mail-in to laboratory.	Cost estimate from Medicare reimbursement[3]<br><br>Code: 91110, 91113<br>Year: 2025<br><br>Included in costs is interpretation by physician and receiver equipment use.<br><br><u>Additional requirements:</u><br>Visit to clinic and interpretation of results by a physician.	<u>Device cost estimate:</u><br>Device capsule: $0.10<br>Electrodes and sensing materials: $0.75<br>Electronics (ex. battery, microcontroller with bluetooth, circuit boards): ~$6.00<br><br><u>Additional requirements:</u><br>Would require a smart-phone or receiver and an algorithm to receive and interpret data
**$0.38**	**~$19**	**~$700-900**	**~$7**

[1] An estimate for a device similar to that reported by Gopalakrishnan, S. *et al.* Smart capsule for monitoring inflammation profile throughout the gastrointestinal tract. *Biosensors and Bioelectronics: X* **14**, 100380, (2023).
[2] Centers for Medicare and Medicaid Services, Clinical Laboratory Fee Schedule, 2025
[3] Centers for Medicare and Medicaid Services, Physician Fee Schedule, 2025

Supplemental Methods
SAS Code used for Figure 5D

```
DATA Biosensors; INPUT  Rat Health &$16. Blue &$ Days; Lines;

1  Healthy  Y  2
1  Healthy  N  1
1  Healthy  N  1
1  DSS  Y  1
1  DSS  Y  1
1  DSS  Y  1
2  Healthy  Y  2
2  Healthy  Y  2
2  Healthy  N  1
2  DSS  Y  1
2  DSS  Y  2
2  DSS  Y  1
3  Healthy  N  1
3  Healthy  N  1
3  Healthy  Y  1
3  DSS  Y  3
3  DSS  Y  1
3  DSS  N  2
4  Healthy  N  1
4  Healthy  N  1
4  Healthy  N  1
4  DSS  N  1
4  DSS  Y  1
4  DSS  Y  1
5  Healthy  N  1
5  Healthy  N  2
5  Healthy  N  1
5  DSS  Y  2
5  DSS  N  1
5  DSS  N  3
6  Healthy  N  3
6  Healthy  Y  1
6  Healthy  N  1
6  DSS  Y  2
6  DSS  Y  2
6  DSS  Y  1
;

RUN;

PROC GENMOD  DATA=Biosensors DESC;
CLASS Rat Health;
MODEL  Blue =  Health Days/ DIST=Binomial LINK=Logit ALPHA=0.05 type3 scale=pearson;
repeated   subject=Rat ;
lsmeans Health / ilink cl;

RUN;
```
